# The influenza pandemic of 1918–1919 in Sri Lanka: its demographic cost, timing, and propagation

**DOI:** 10.1111/irv.12238

**Published:** 2014-02-24

**Authors:** Siddharth Chandra, Dilshani Sarathchandra

**Affiliations:** a Asian Studies Center, Michigan State UniversityEast Lansing, MI, USA; b Department of Sociology, Michigan State UniversityEast Lansing, MI, USA

**Keywords:** 1918–1919 influenza pandemic, Ceylon, epidemic, influenza, island, mortality, spread, Sri Lanka

## Abstract

**Background:**

As an island and a former British colony, Sri Lanka is a case of special interest for the study of 1918–1919 influenza pandemic because of its potential for isolation from as well as integration into the world epidemiologic system.

**Objectives:**

To estimate population loss attributable to the influenza pandemic and weekly district-level excess mortality from the pandemic to analyze its spread across the island.

**Methods:**

To measure population loss, we estimated a population growth model using a panel of 100 district-level observations on population for five consecutive censuses from 1891 to 1931, allowing for a one-time drop in population in 1918–1919. To estimate weekly excess mortality from the pandemic, we estimated a seasonally adjusted weekly time series of district-specific mortality estimates from vital registration records, ranked them, and plotted the ranks on weekly maps to create a picture of the geographic pattern of propagation across Sri Lanka.

**Results:**

Total loss of population from the influenza pandemic was 307 000 or approximately 6·7% of the population. The pandemic peaked in two discrete (northern and southern) regions in early October of 1918 and in a third (central) region in early March 1919.

**Conclusions:**

The population loss estimate is significantly higher than earlier estimates of mortality from the pandemic in Sri Lanka, suggesting underreporting of influenza-attributable deaths and a role for influenza-related fertility declines. The spatial pattern of peak mortality indicates the presence of two distinct entry points and three distinct epidemiologic regions, defined by population density and ethnicity, in colonial Sri Lanka.

## Introduction

The influenza pandemic of 1918–19 was one of the most destructive global epidemics in history. The H1N1 family of influenza viruses has, moreover, been responsible for the largest number of deaths in human history.^1^ Estimates of mortality from the pandemic range from 20 to 40 million,^2^ 20–50 million,^3^ 40–50 million,^4^ and 50–100 million^5^ and include a variety of point estimates, including 20 million,^6^ 30 million,^7^ and 40 million.^8^–^10^ As an island nation and a former British colony, Sri Lanka, then known as Ceylon, is a case of special interest because of its potential for isolation from as well as integration into the world epidemiologic system. During the global pandemic of 1918–1919, influenza was first reported in Sri Lanka in June 1918.^11^–^13^ However, mortality from influenza increased noticeably only in September 1918,^14^ peaking in most places in the last quarter of that year. According to the Registrar-General of Ceylon, ‘[n]o epidemic within recent years has been so widespread and so fatal as “influenza,” which was raging in the Island during the latter half of 1918’.^14^ Prior estimates of mortality from the 1918–1919 influenza pandemic in Sri Lanka range from 41 916 to 91 600. The Registrar-General for Ceylon reported 41 916 deaths due to influenza (excluding pneumonia and other complications) in 1918–1919.^14^ At the time, the number of deaths registered in 1918 was ‘the highest ever recorded in Ceylon in any single year’, and ‘this comparatively high mortality is due to the influenza pandemic’.^14^ ‘The death rate per million of the estimated population in 1918, viz., 4084, was 170 times as high as in 1917, when the rate was only 24’.^14^ In 1919, influenza again took a heavy toll, with ‘no fewer than 22 814 deaths being attributed to this cause’.^15^ Langford and Storey^12^ estimated approximately 50 000 deaths due to influenza, Johnson^16^ estimated 91 600 deaths, and Lee *et al*.^17^ reported a range of 51 000–91 600 deaths. The Census of Ceylon estimated excess mortality from influenza in 1918–1919 at 57 000.^18^

The epidemiology of the 1918–1919 influenza pandemic in Sri Lanka was similar to its epidemiology elsewhere. First, it occurred in at least two distinct waves^19^ as it had done in a number of other countries, including neighboring India^20^,^21^ and Indonesia^22^ in Asia; Scotland, England and Wales,^16^ and Portugal and Spain^23^ in Europe; Mexico^24^ and Peru^25^ in the Western Hemisphere; and cities including New York City^26^ and Copenhagen.^27^ The first and relatively mild wave occurred in the summer of 1918, only to be followed by a more severe second wave in the autumn of 1918 and a third wave in the spring of 1919.^12^ The second wave was far more virulent and widespread than the first wave, and lasted from October to December 1918, with pockets continuing into January and even February 1919 (Ibid).

In terms of its propagation, the Principal Civil Medical Officer of Ceylon noted that the capital city, Colombo, which was also a major port, was the entry point of the disease.^12^ Langford and Storey^12^ speculate, however, that influenza entered Sri Lanka through *two* separate locations, Colombo in the south and Talaimannar in the northwest. Coastal districts in the north, northeast and west and districts in the southwest near Colombo were affected initially. Subsequently, the disease spread to the interior of the island and to other districts in the south.^12^ The final district to be affected by influenza was Batticaloa on the east coast (Ibid). The second part of this paper, which focuses on the pattern of propagation of the peak wave of influenza across Sri Lanka, will revisit and build on this account.

## Methods

In the first part of the analysis, following Davis,^28^ this paper utilizes the population loss method to evaluate the demographic cost of the influenza pandemic on the population of Ceylon. Briefly, the population loss method involves using decennial census data from multiple censuses of Ceylon to estimate the trajectory of population before and after the pandemic. By allowing for a one-time break in this trajectory corresponding to 1918–1919, it is possible to estimate the loss in population, net of the normal growth trajectory, coincident with the influenza pandemic. For this purpose, we collected 100 district-level observations on population for five consecutive censuses from 1891 to 1931. In the absence of accurate statistics on influenza-attributable mortality, the relatively accurate total population counts in the colonial censuses can provide us with a meaningful estimate of the overall demographic impact of the pandemic.

Standard population growth trajectories are estimated using panel data regression methods. The models, developed in Chandra *et al.,*^29^ are specified as follows:


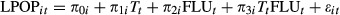


The following description closely follows Chandra *et al*. (Ibid). The dependent variable is the log of population in district *i* in year t, *T*_*t*_ is a time trend that accounts for the year-on-year growth of population, FLU_*t*_ is a dummy variable that captures a one-time change in population during the influenza pandemic, and *ɛ*_*it*_ is the random error term. The parameters denoted by *π*_0*i*_ – *π*_3*i*_ capture district-specific intercepts, the population growth rate prior to the pandemic, the one-time shift in the growth curve during the pandemic, and the change in the population growth rate after the pandemic, respectively. These parameters can be estimated as fixed effects or random effects. Both specifications were estimated using the sas software,^30^ and standard Hausman and Breusch Pagan tests to select between these two methods were conducted. Based on these tests, we selected the random effects specification. In addition, we estimated models in which the population growth rate after the pandemic was allowed to differ from the growth rate before the pandemic (‘unrestricted’ models) as well as models in which these two growth rates were constrained to be the same (‘restricted’ models). We also estimated and compared the population growth trajectories for the entire set of districts in the dataset with those for two subsets of districts (one including the heavily populated and migration-intensive Kandy district and the other excluding it) for which migration was not an important driver of population change.

We also estimated models with spatially correlated errors. Population change estimates varied by less than 1%. In a small number of cases, the estimation algorithm for models with spatial autocorrelation did not converge (which they did for all models not containing the spatial autocorrelation assumption). For these reasons, we decided to retain models that have spatially uncorrelated errors.

**Figure 1:**
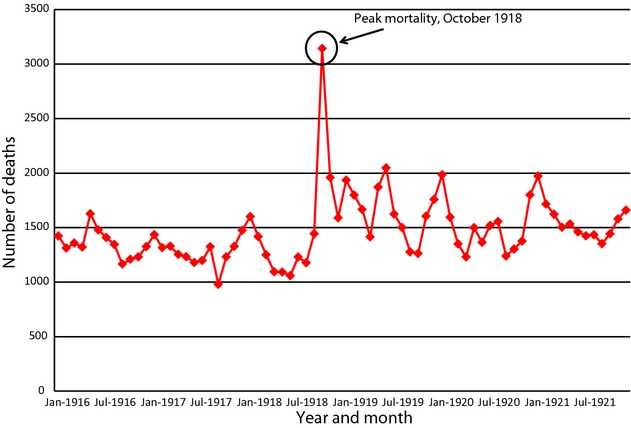
Deaths registered in Colombo District, 1916–1921.

The second part of the analysis aimed at creating a picture of the spread of the pandemic across Ceylon. This analysis was motivated by the observation that while monthly influenza-attributable mortality was not reported, total monthly mortality statistics for the districts, reported in the Registrar-General's reports, provide evidence about this phenomenon. Specifically, aggregate monthly district-level mortality for late 1918 and early 1919 is noticeably higher than monthly mortality for any other time during the 6-year period from January 1916 to December 1921 (see Figure 1, for Colombo, for example, for which there is a clear peak in October 1918). Taking this observation as a starting point and recognizing that mortality from a variety of other causes, many of them seasonal, influence aggregate mortality, we first seasonally adjusted the district-level mortality statistics using the PROC X12 procedure in sas.^31^ This procedure has the effect of producing time series data on mortality after the effects of seasonal diseases, including malaria, cholera, and other major drivers of mortality, have been eliminated. The seasonally adjusted monthly mortality data were then interpolated to produce estimates of seasonally adjusted weekly district mortality for the period January 1916 to December 1921 (Ibid, EXPAND procedure). Next, because the focus of this analysis was on the timing and spread of influenza and the mortality statistics themselves are imprecise and probably underestimate actual mortality because of the persistent problem of underreporting, rather than using the mortality numbers themselves, we converted these seasonally adjusted data into weekly ranks, paying attention not to the excess reported mortality in each district, but rather to the intensity of mortality during each week relative to other weeks in the dataset. For each district, the week with the highest seasonally adjusted mortality was ranked 1, the week with the second highest mortality was ranked 2, and so forth. Therefore, for each district, the rank represents the rank of the week in terms of numbers of deaths (after seasonal adjustment) compared to all other weeks (approximately 300) for that district over the 6-year period from 1916 to 1921. If two districts have the same rank of, say, 2, this means that both districts experienced their second highest level of weekly mortality in the 5-year interval of 1916–1921 during that week. These two districts do not necessarily have the same mortality rates as each other.

For each week, a district map of Ceylon (reflecting the colonial district configuration) was plotted, and districts for which mortality was in the top 4 weeks of the 314 possible weeks in the sample were highlighted in color.

## Results and discussion

**Figure 2:**
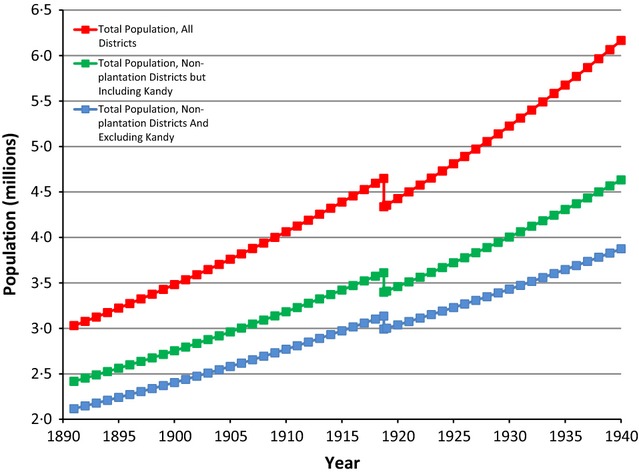
Population trend for Sri Lanka showing population loss due to the influenza pandemic of 1918–1919.

The results of the two sets of analyses are striking. In some cases, they conform to earlier held notions about the demographic impact, timing, and geographic spread of the pandemic. In others, they suggest that a significant revision of our knowledge of the pandemic is necessary. Table 1 contains the estimates of the population trajectory model described above, and Figure 2 illustrates this trajectory for all the districts of Sri Lanka and for a subset for which migration was not an important determinant of population change. A number of interesting new findings include first a coefficient associated with the influenza pandemic that is sizeable, negative, and statistically significant, at −0·1079 in the restricted model (Table 1, Column 2). This translates into an estimated population loss of 307 000 (±86 000, α = 0·10), which is significantly in excess of the highest mortality estimate of 90 000. Because this large population loss figure incorporates losses due to the direct effect of influenza as well as its indirect effects, including depressed fertility, possible post-pandemic starvation, and complications from influenza including pneumonia, it provides a more comprehensive measure of the total demographic cost of the event. In addition, given the magnitude of the estimate of total population loss, it is also likely that the mortality figure itself was larger than the standing estimate of 90 000. Indeed, the Registrar-General's reports specifically mention some of these indirect effects. On birth rates, the report of 1919 notes that ‘[t]he rate for the third quarter, which is usually the lowest, shows a more marked decline this year than in any of the preceding 10 years. This low rate is undoubtedly the aftermath of the influenza epidemic’.^15^ Similarly, on pneumonia, an often fatal complication of the influenza, the report for 1918 states ‘…the extent of the death roll due to the epidemic cannot be measured from the statistics of influenza alone, as in a very large number of cases the deaths may have been attributed to pneumonia both by qualified medical practitioners and village registrars. This is borne out by the large increase in the deaths from pneumonia…'.^14^

**Table 1:** Population growth models with influenza mortality estimates

Estimates	All districts		Non-plantation districts but including Kandy		Non-plantation districts and excluding Kandy	
		
	Model specification		Model specification		Model specification	
		
	Unrestricted	Restricted	Unrestricted	Restricted	Unrestricted	Restricted
Intercept (γ_00_)	−17·6389***	−18·1941***	−15·8671***	−15·9297***	−15·3448***	−14·3955***
	*0·0001*	*0·0001*	*0·0001*	*0·0001*	*0·0001*	*0·0001*
Time trend (γ_10_)	0·0154***	0·0157***	0·0145***	0·0145***	0·0142***	0·01365***
	*0·0001*	*0·0001*	*0·0001*	*0·0001*	*0·0001*	*0·0001*
Flu dummy (γ_20_)	−0·1093***	−0·1079***	−0·1221***	−0·1219***	−0·1069***	−0·1095***
	*0·0001*	*0·0001*	*0·0003*	*0·0002*	*0·0014*	*0·0011*
Flu dummy*Time trend (γ_30_)	0·0011	–	0·0001	–	−0·0020	–
	*0·5678*	–	*0·9522*	–	*0·0651*	–
Number of observations	100		75		70	
Akaike's Information Criterion	−53·6	−64·0	−36·3	−46·9	−46·4	−55·9
Estimates of key demographic phenomena						
Influenza population loss (‘000)	313	307	215	214	143	150
Population change, 1918 to 1919 (‘000)	241	236	163	152	101	108
Annual population growth rate to pandemic	1·54%	1·57%	1·45%	1·45%	1·42%	1·37%
Annual population growth rate after pandemic	1·65%	1·57%	1·46%	1·45%	1·22%	1·37%

*P*-values for null hypothesis of 0 coefficient in italics.

*** *P* < 0·01.

A second finding relates to the rate of population growth in Ceylon. The unrestricted and restricted models suggest that this rate was between 1·54% and 1·65% per year before and after the pandemic. This finding of uniform population growth mirrors similar findings for Indonesia and Japan,^32^,^33^ but stands in contrast to the recent finding for India,^29^ that the rate of population growth after the pandemic was significantly higher than that before the pandemic. Increasingly, it appears that steady pre- and post-pandemic rates of population growth were the norm in Asia and that India was an exception.

**Figure 3:**
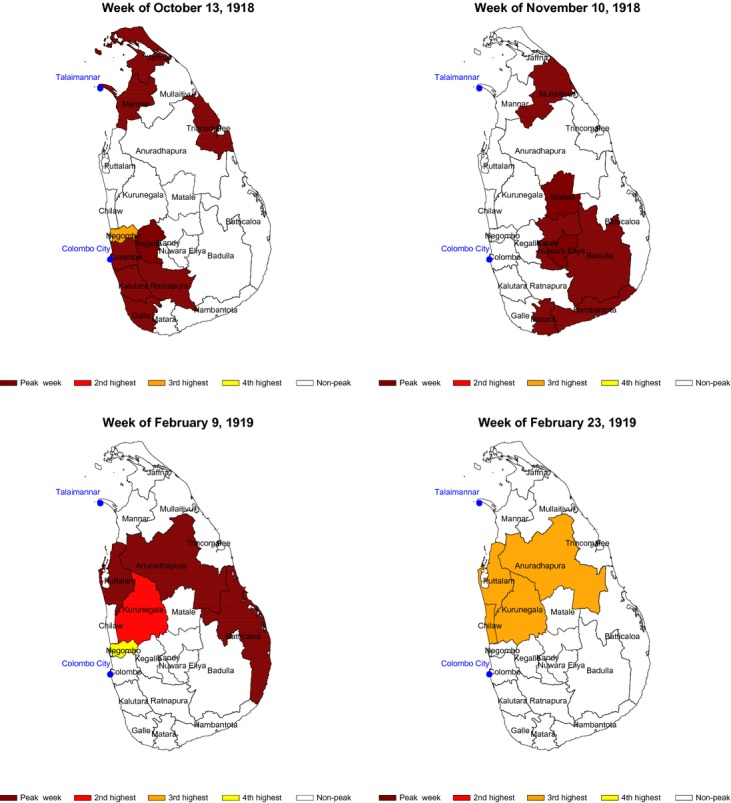
Districts with peak mortality during the influenza pandemic of 1918–1919.

Equally interesting are the findings on the spread of the pandemic across the island. Keeping in mind that the analysis emphasizes periods of peak mortality, the pattern is as follows. Mortality peaked almost simultaneously in the northern and southern parts of Ceylon, in September 1918 (Figure 3). In terms of its progress across the northern and southern regions, the epidemic appears to have taken a west-to-east direction. These two observations are consistent with Langford and Storey's^12^ speculation that the influenza entered Sri Lanka simultaneously from two points — Colombo on the southwestern coast and Talaimannar on the northwestern coast. Both were busy ports handling high volumes of international passenger traffic, with Colombo linking Sri Lanka to a variety of ports around the world and Talaimannar linking Sri Lanka to southern India.

The analysis of peak mortality times also reveals a phenomenon that is distinctive for Sri Lanka. That is, while the epidemic in the north and south of the island peaked in the autumn of 1918, it was not until a few months later, in the spring of 1919, that a number of districts in the central part of the island experienced their peak mortality (Figure 3). This point, which is noted by Langford and Storey, suggests a relative isolation of populations in the central districts of the island from the north and the south, as well as the isolation of the north and the south from each other. This is in stark contrast with the pattern observed elsewhere, where the disease moved wavelike across entire countries. A second interesting (and hitherto unknown) feature of the peak pattern of mortality in these central districts is that the peak period in the west was longer in duration than the peak period in the east, a pattern that was not observed in the northern and southern regions (Figure 3). The accompanying animated slide show (Appendix S1) illustrates the spatiotemporal nature of the progress of the pandemic across Sri Lanka.

### Limitations

The results presented in this paper should be viewed keeping in mind several limitations, relating primarily to the historical nature of the data used in the analysis. First, the measurement of the demographic cost of the pandemic relies on models estimated using decennial census data on population, and interpolating them to find the loss in population in 1918–1919. The study of the propagation of the influenza likewise uses interpolation to obtain weekly estimates from monthly data. In both cases, the sets of estimates and patterns produced are quite plausible, suggesting the appropriateness of the use of interpolation. In the case of population loss, the rates lie within the range of population loss estimated for other countries in the region.^32^,^33^ In the case of propagation, the natural west-to-east district-to-adjacent-district movement of the pandemic is consistent with what one might expect. A second limitation is the absence of direct measures of influenza mortality or infections, either or both of which would have contributed to more precise estimates and patterns. For this reason, it was necessary to deploy the population loss method to generate Sri Lanka wide estimates of population loss, and to subject monthly all-cause mortality data to seasonal adjustment in order to flush out the effects of important seasonal diseases such as cholera and malaria from the data. That said, the propagation results, highlighted in Figure 3, are clearly consistent with and add to the earlier literature on the spread of the disease and with what we know about an infectious disease like influenza.

## Conclusion

The influenza pandemic in Sri Lanka demonstrated some of the features of the 1918–1919 global influenza pandemic observed in other countries. It caused extremely high numbers of deaths on the island and affected different regions in at least two distinct and severe waves. Results of our research suggest a need to revise current estimates of influenza mortality in Sri Lanka due to the pandemic. In contrast with the existing high estimate of 91 600 deaths or 1·1% of the population,^12^ our point estimates of loss of population are 307 000 (± 86 000, α=0·10) and 313 000 (± 91 000, α=0·10) or 6·7% of the population. This finding calls into question the assertion that influenza mortality in Sri Lanka was ‘very much lighter than the mortality in India where according to Mills (1989:256) almost 5·5 percent of the population died’.^12^ Our results show that the demographic impacts of the influenza pandemic in Sri Lanka were similar to and as severe as those of neighboring India.

In terms of geography, the findings build on the earlier work of Langford and Storey.^12^ They confirm that the pandemic most likely entered Sri Lanka through Colombo in the south and support their suggestion about a parallel entry point in Mannar in the northwest. The pandemic first peaked in these two coastal port areas and then radiated from the west to the east. These episodes of peak mortality lasted about 8 weeks from early October to early December of 1918. A number of districts in the central part of the island were not part of this dynamic. These central districts experienced a wave of peak mortality that lasted about 6 weeks from early February to mid-March of 1919 and that differed from the earlier wave in terms of its duration in the different districts that were affected by it. These findings suggest the existence of three separate epidemiologic zones in colonial Sri Lanka for influenza and perhaps even other diseases that followed a person-to-person mode of transmission. These three zones more or less coincided with patterns of ethnic distribution and population density; the largest ethnic group, the Sinhalese, were found chiefly in the central, western and southern parts of the island; ‘Ceylon Tamils’, defined as people of Tamil ethnicity but Ceylonese ancestry, were found mainly in the northern parts of the island; and ‘Indian Tamils’, defined as people of Tamil ethnicity but Indian ancestry, resided in the south-central part of the island.^34^ In addition, the north-central districts, which did not experience peak mortality during the autumn of 1918, were also the districts with the lowest population density.^34^ This feature very likely contributed to a peak mortality pattern that was altogether different from that experienced by the more densely populated northern and southern regions of the island. The spread of the pandemic across the three regions in distinct waves suggests some isolation of the main ethnic groups from one another, and impediments to person-to-person contact across ethnic lines and barriers of low population density.

As far as the interplay between the global influenza pandemic and its occurrence in Sri Lanka is concerned, it appears that the northern and southern zones were directly linked to the global epidemiologic system. The sparsely populated central zone separated the northern and southern zones from each other, appears to have been isolated from the global system, and experienced a dynamic all of its own. This finding further suggests that geographic contiguity alone was not a sufficient condition for the transmission of the mortality wave and that other factors such as person-to-person contact across regions, or the lack of it, may have played an important role in the spread of the disease.^35^ Given the scarcity of studies that examine the spread of the 1918 influenza pandemic at a relatively high level of geographic and temporal resolution, it is hoped that the methods used in this paper will encourage scholars to develop a more complete picture of the pandemic in different locations, thereby illuminating the factors that facilitated or hampered its spread. These insights can then be leveraged to inform strategies to contain similar events in the future using our existing knowledge about the importance of social distancing and other relevant spatial features of populations.

## Supporting Information

**Appendix S1.** Weekly progression of influenza across Sri Lanka during the 1918-1919 pandemic.
