# Supplementary material for: The influenza pandemic of 1918–1919 in Sri Lanka: its demographic cost, timing, and propagation
Source: Influenza Other Respir Viruses. 2014 Feb 24;8(3):267–73. doi: 10.1111/irv.12238 (PMC4181474; doi:10.1111/irv.12238)

## Slide 1
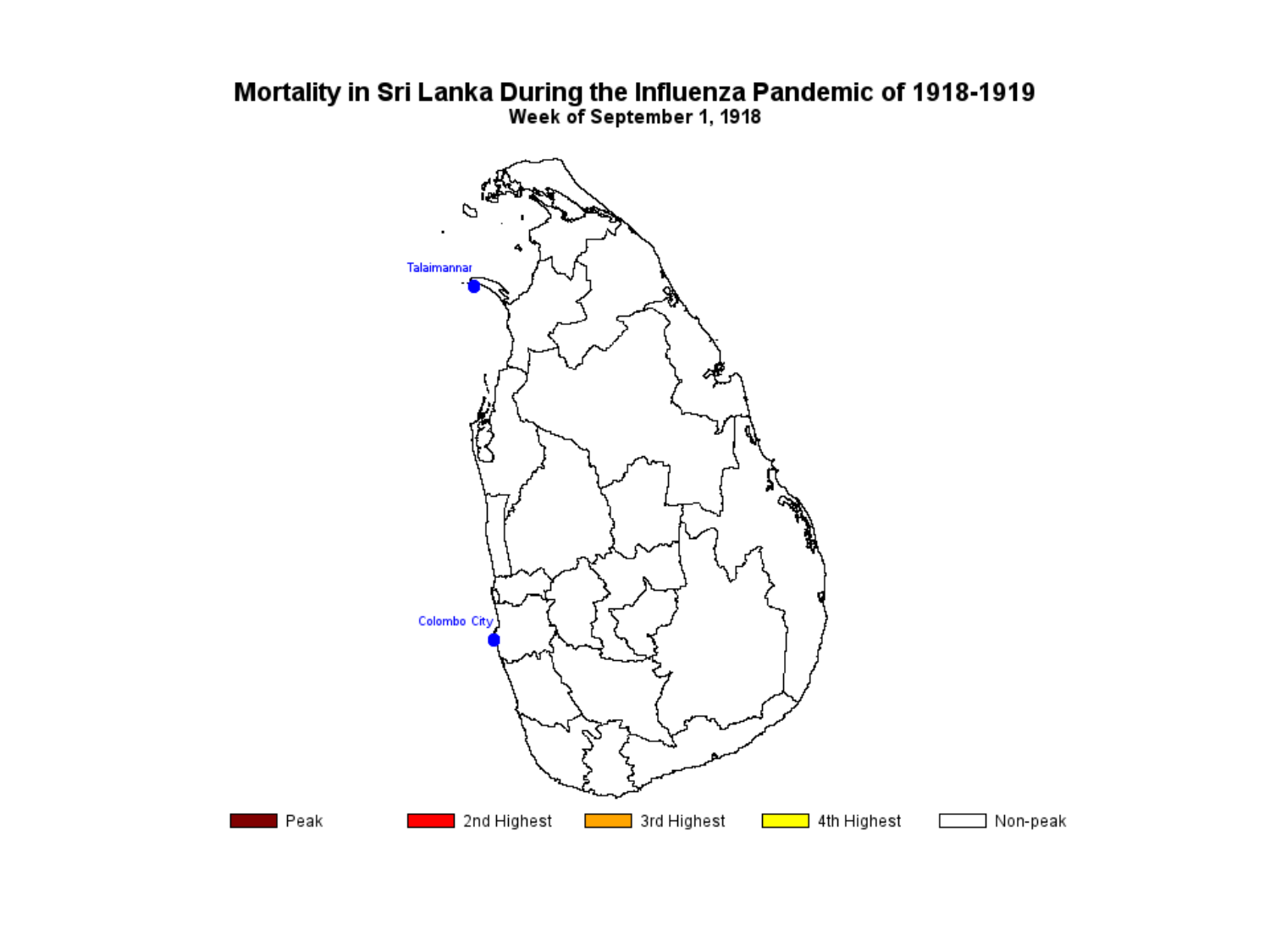

## Slide 2
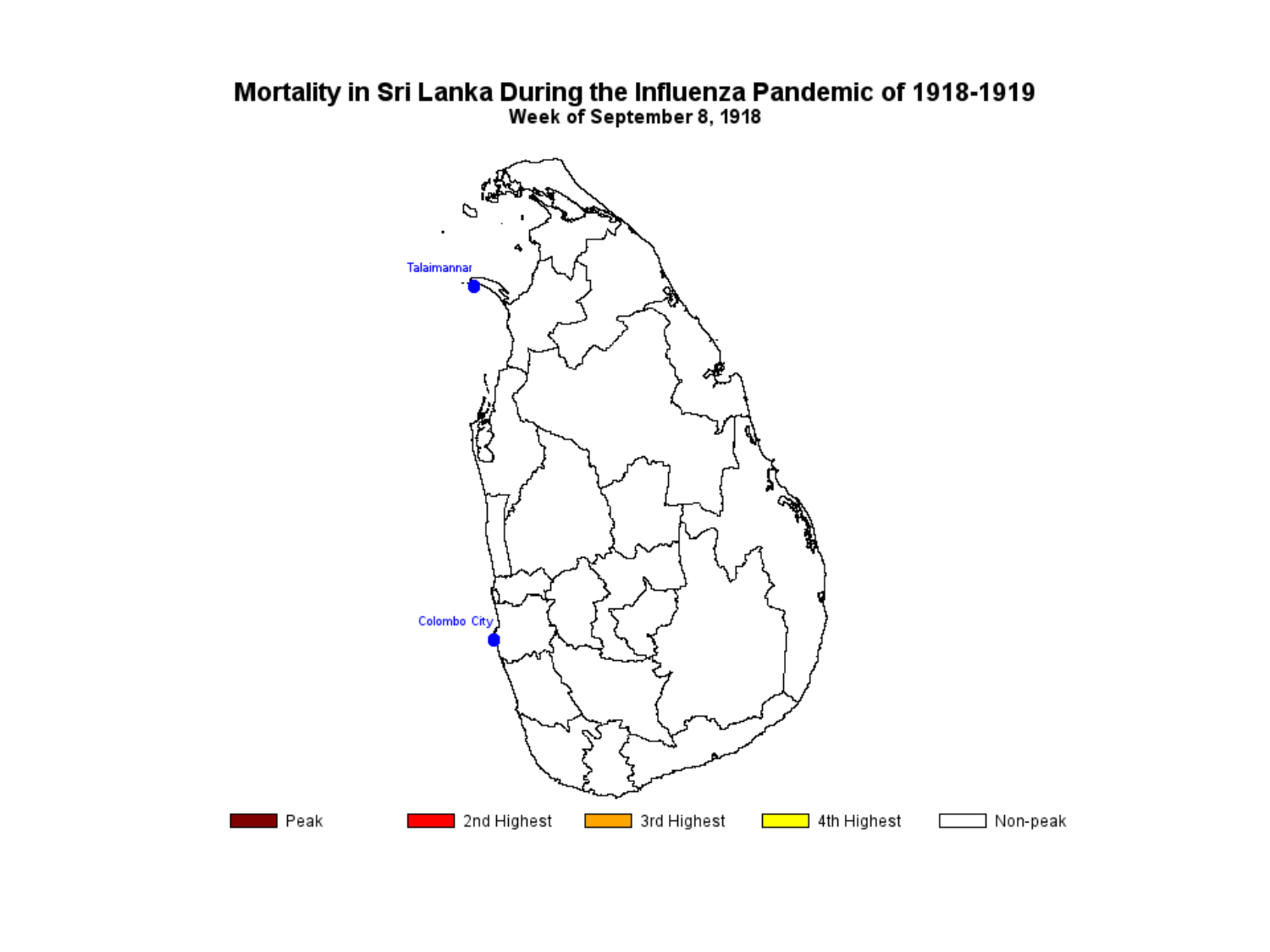

## Slide 3
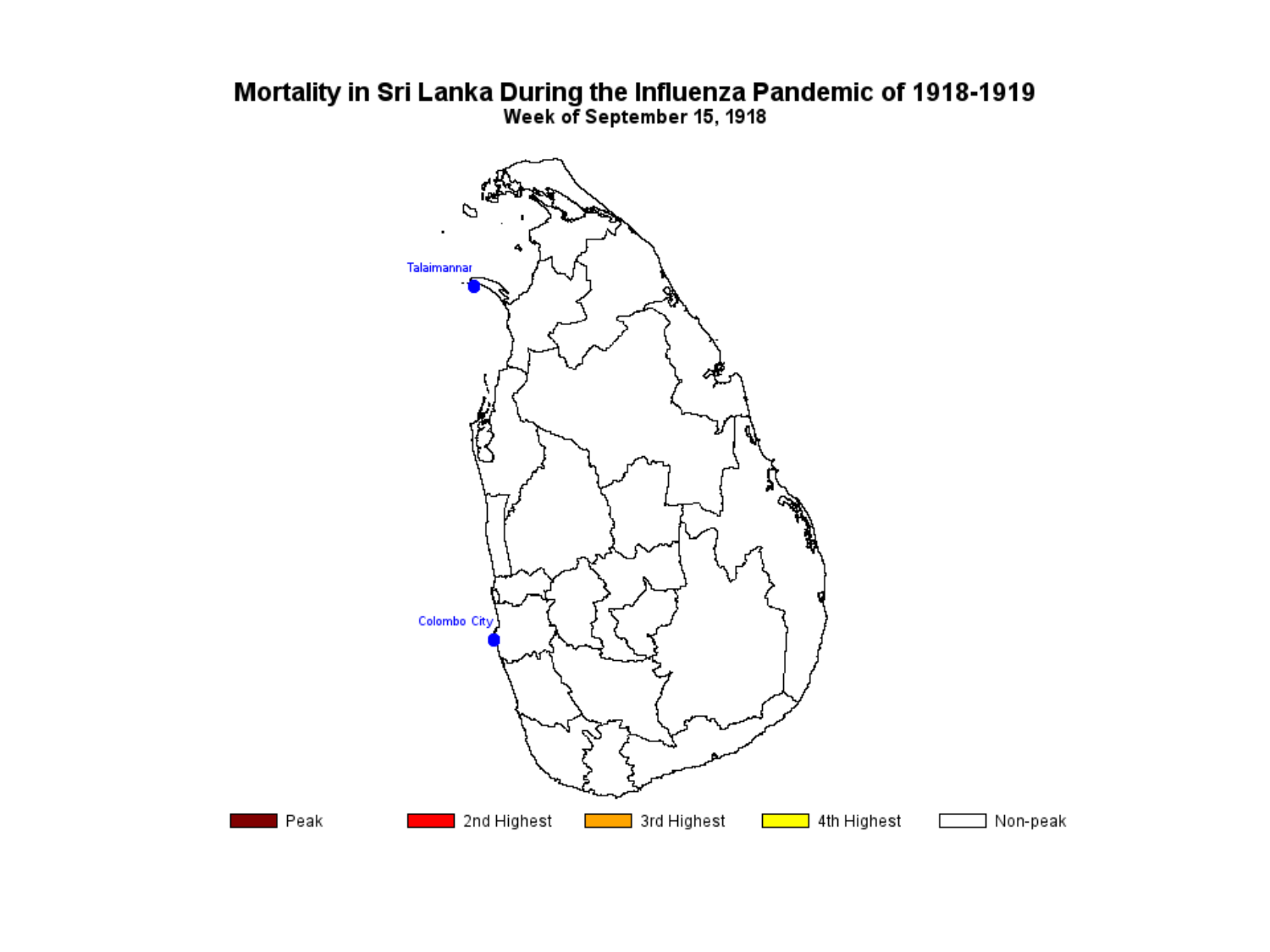

## Slide 4
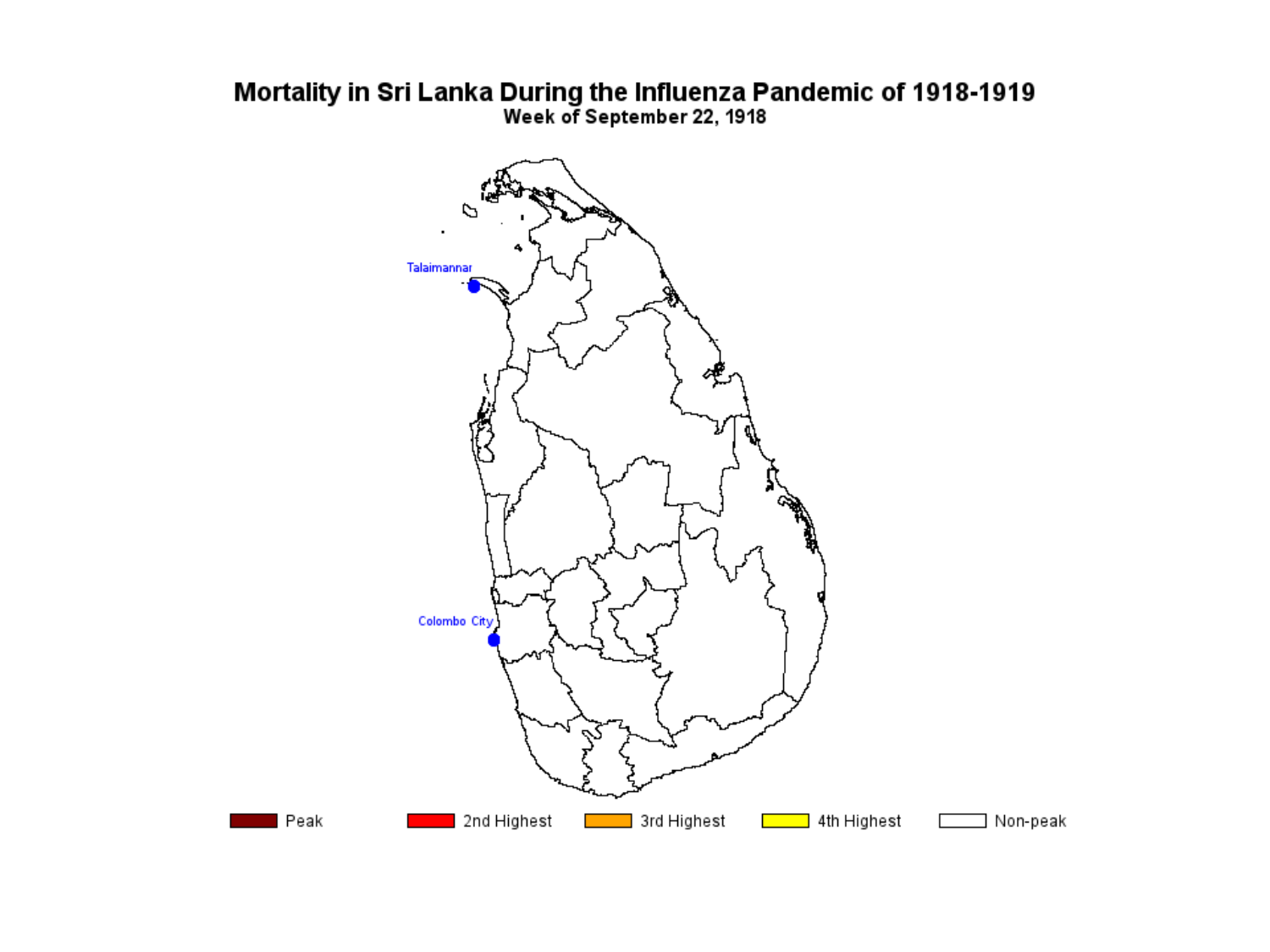

## Slide 5
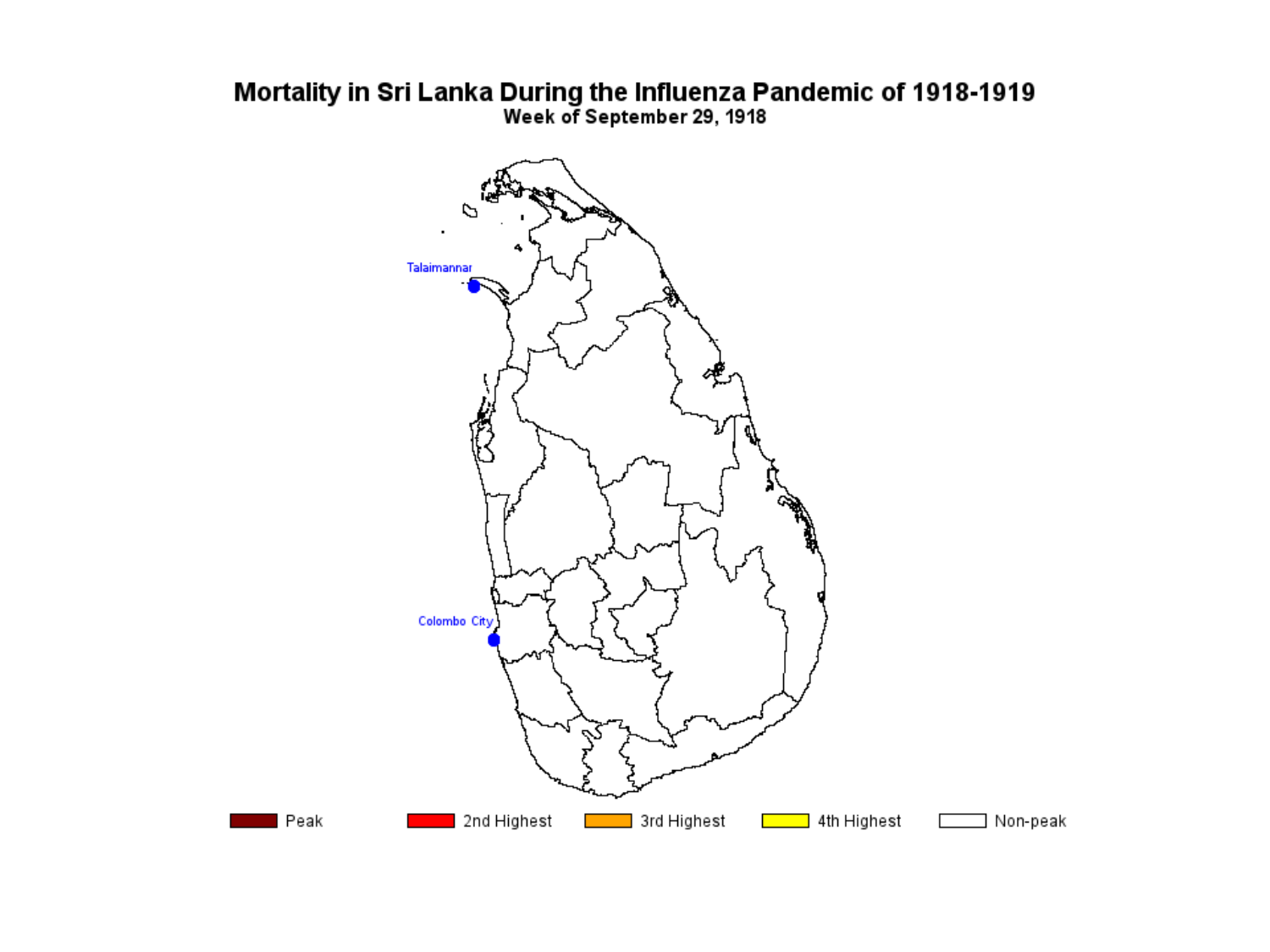

## Slide 6
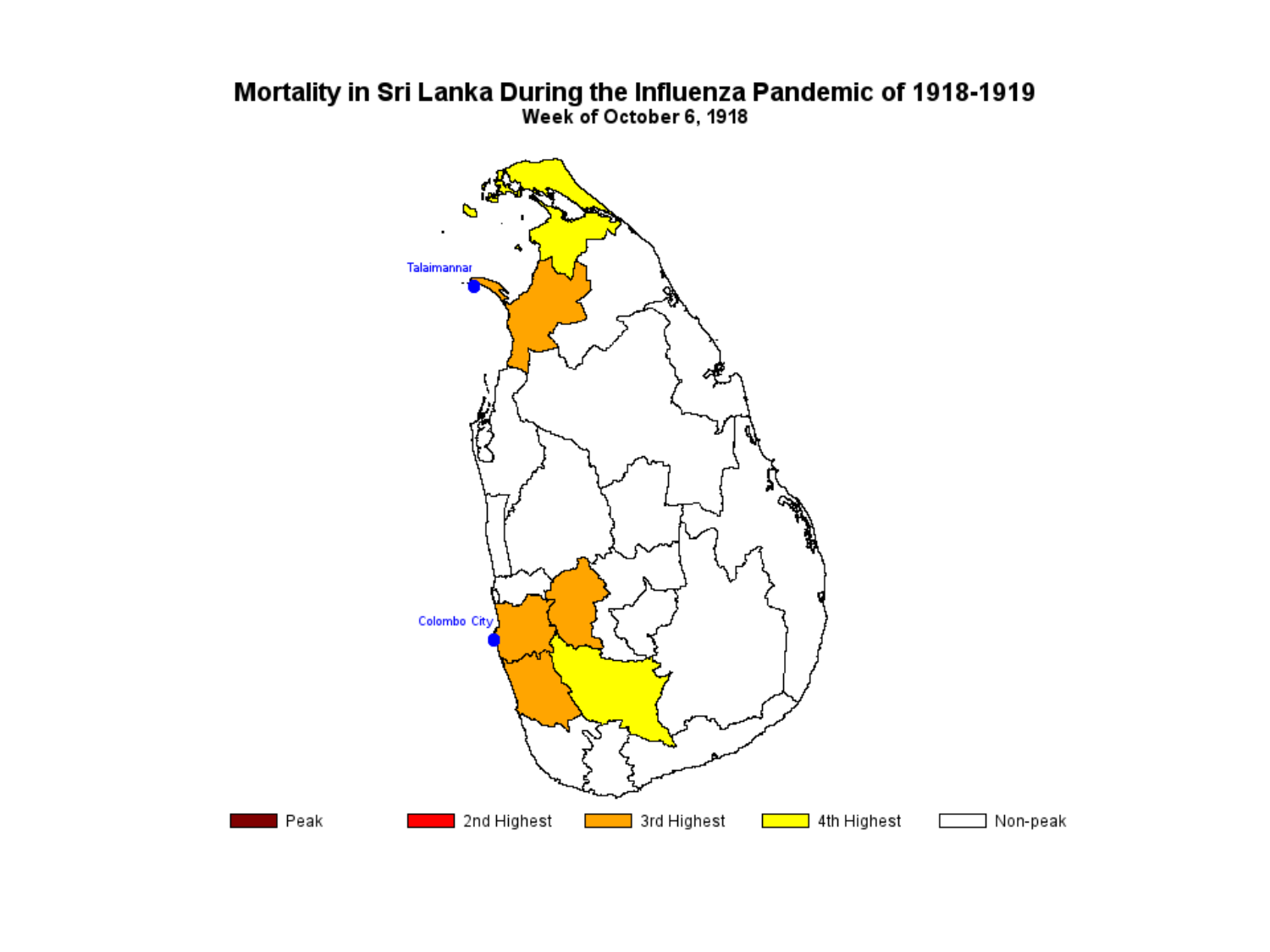

## Slide 7
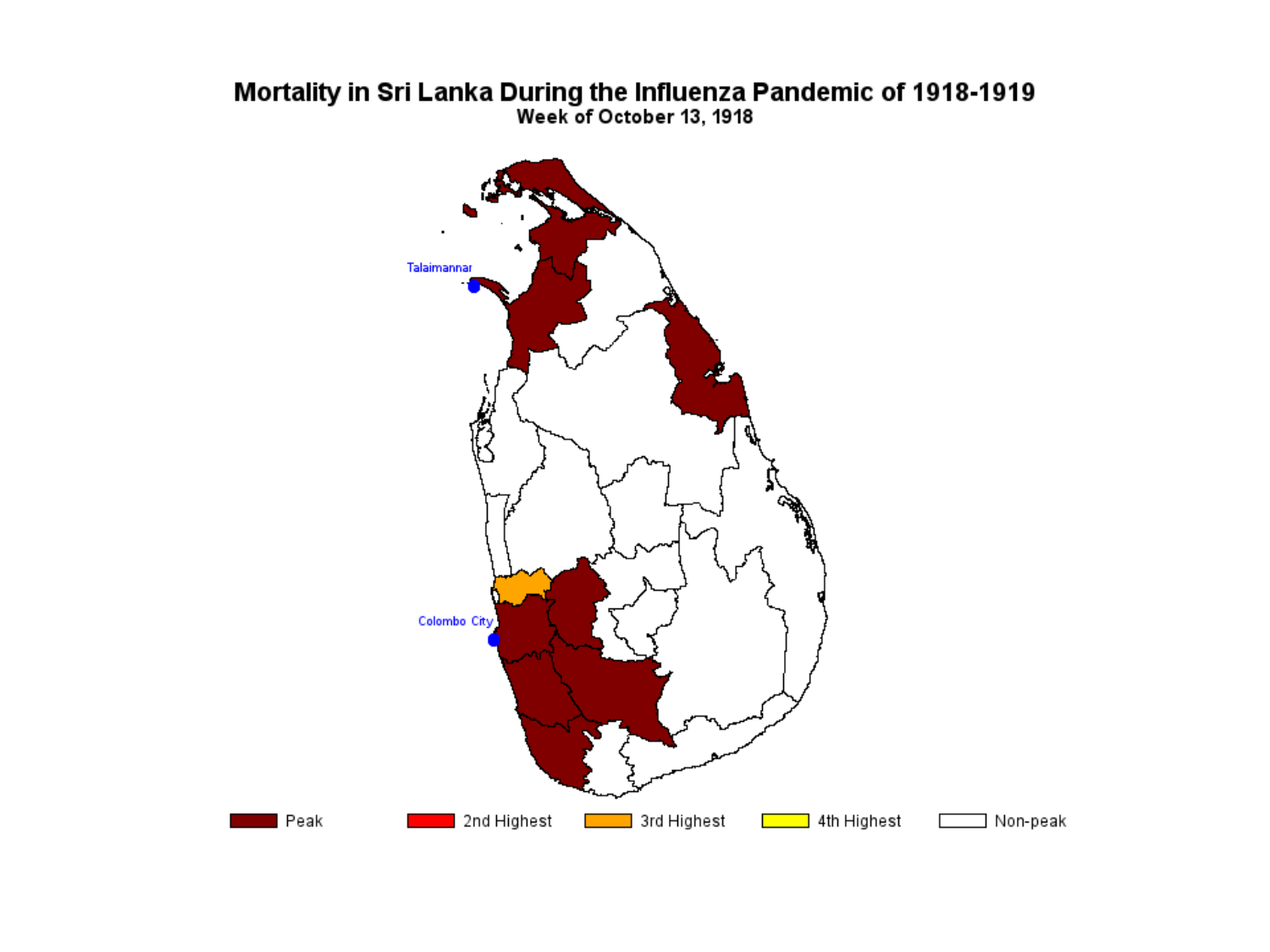

## Slide 8
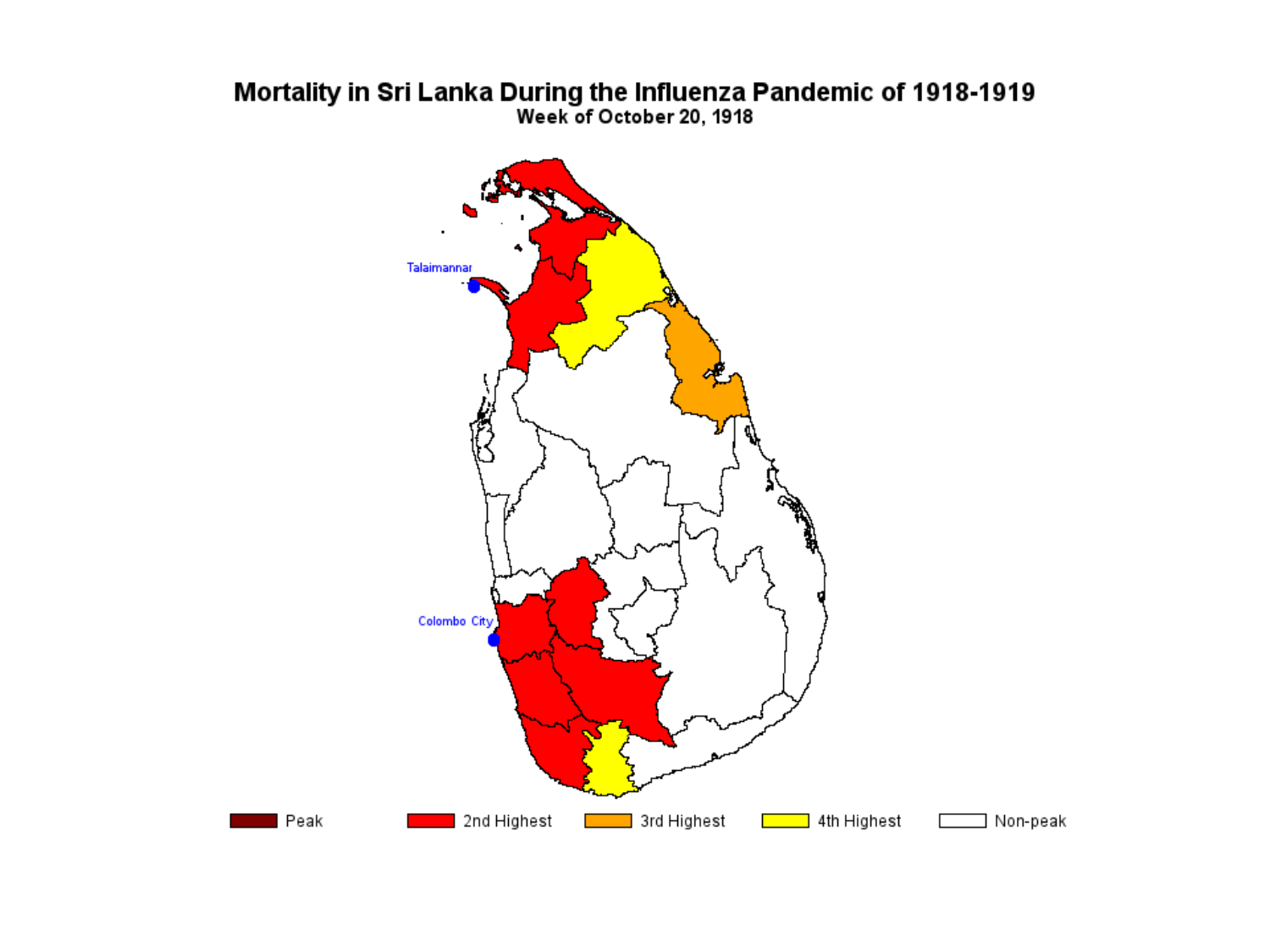

## Slide 9
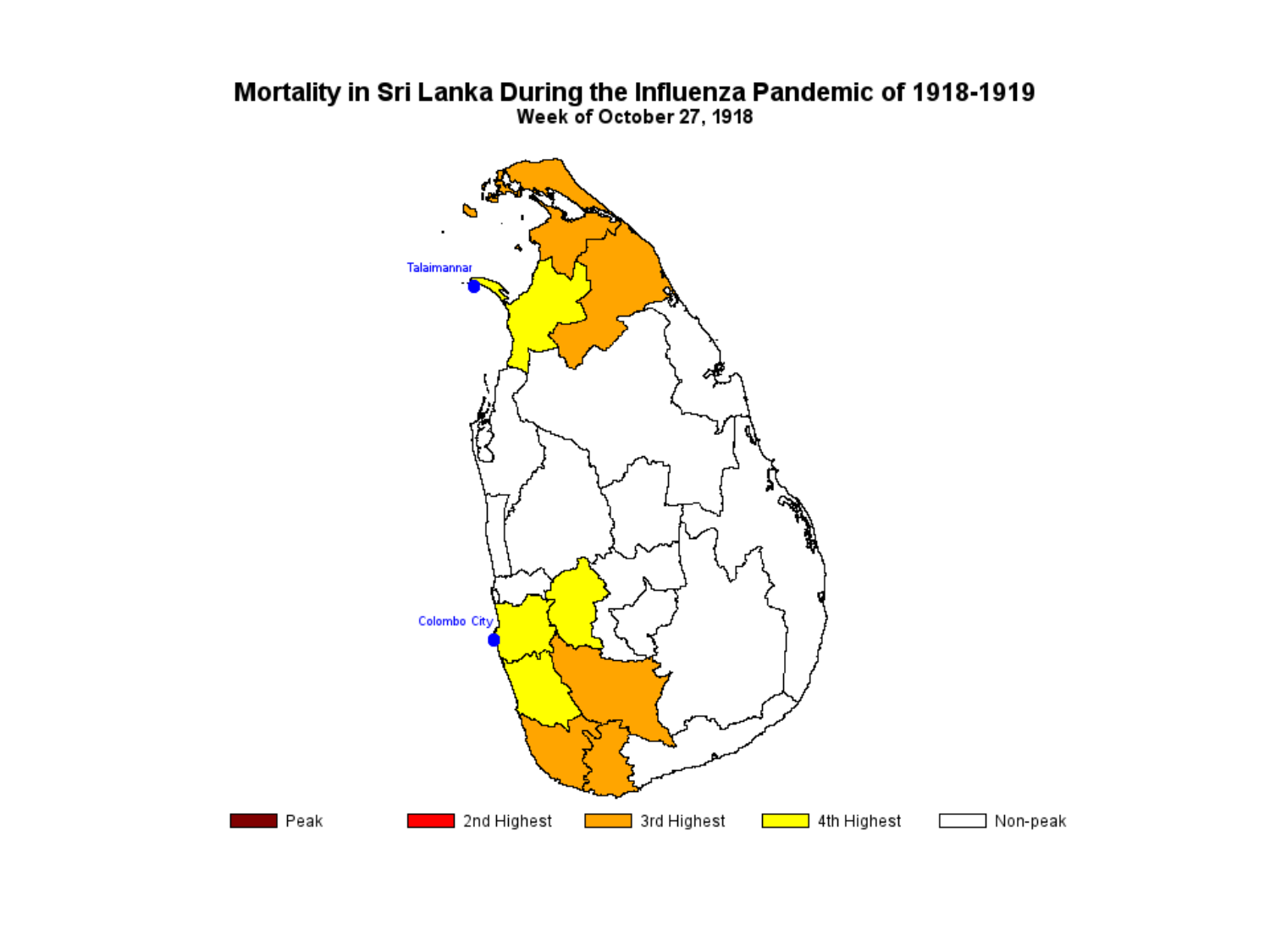

## Slide 10
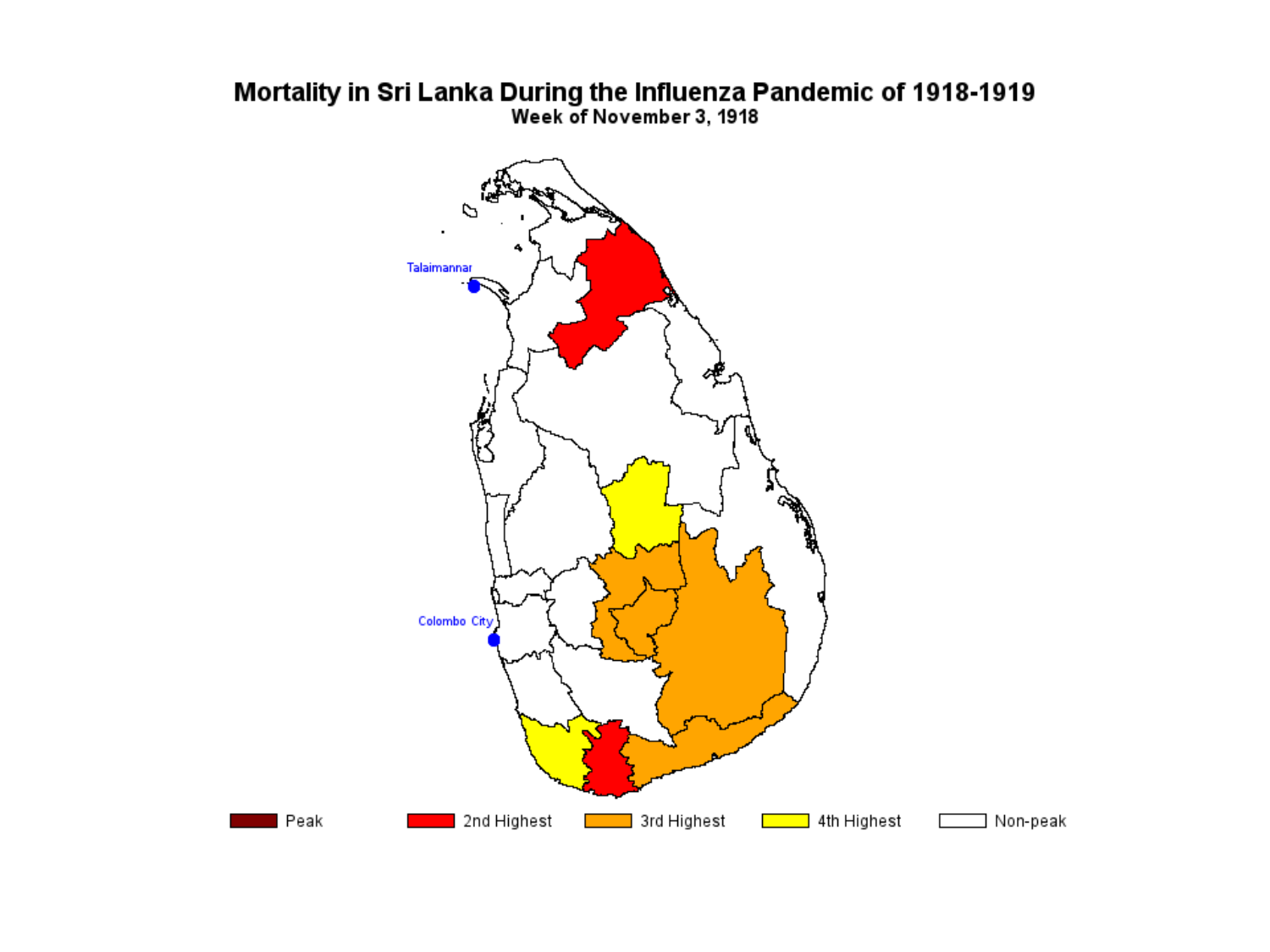

## Slide 11
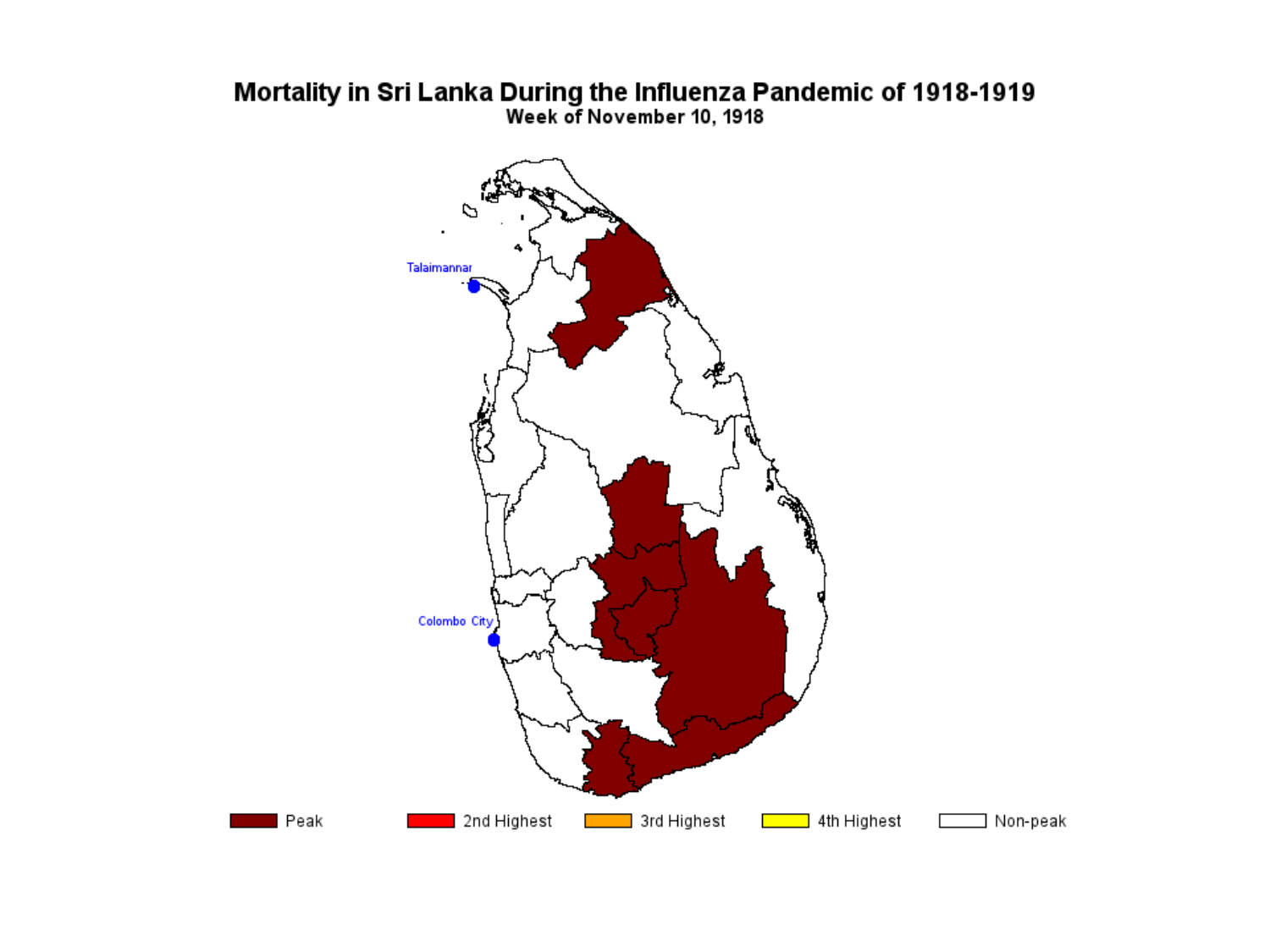

## Slide 12
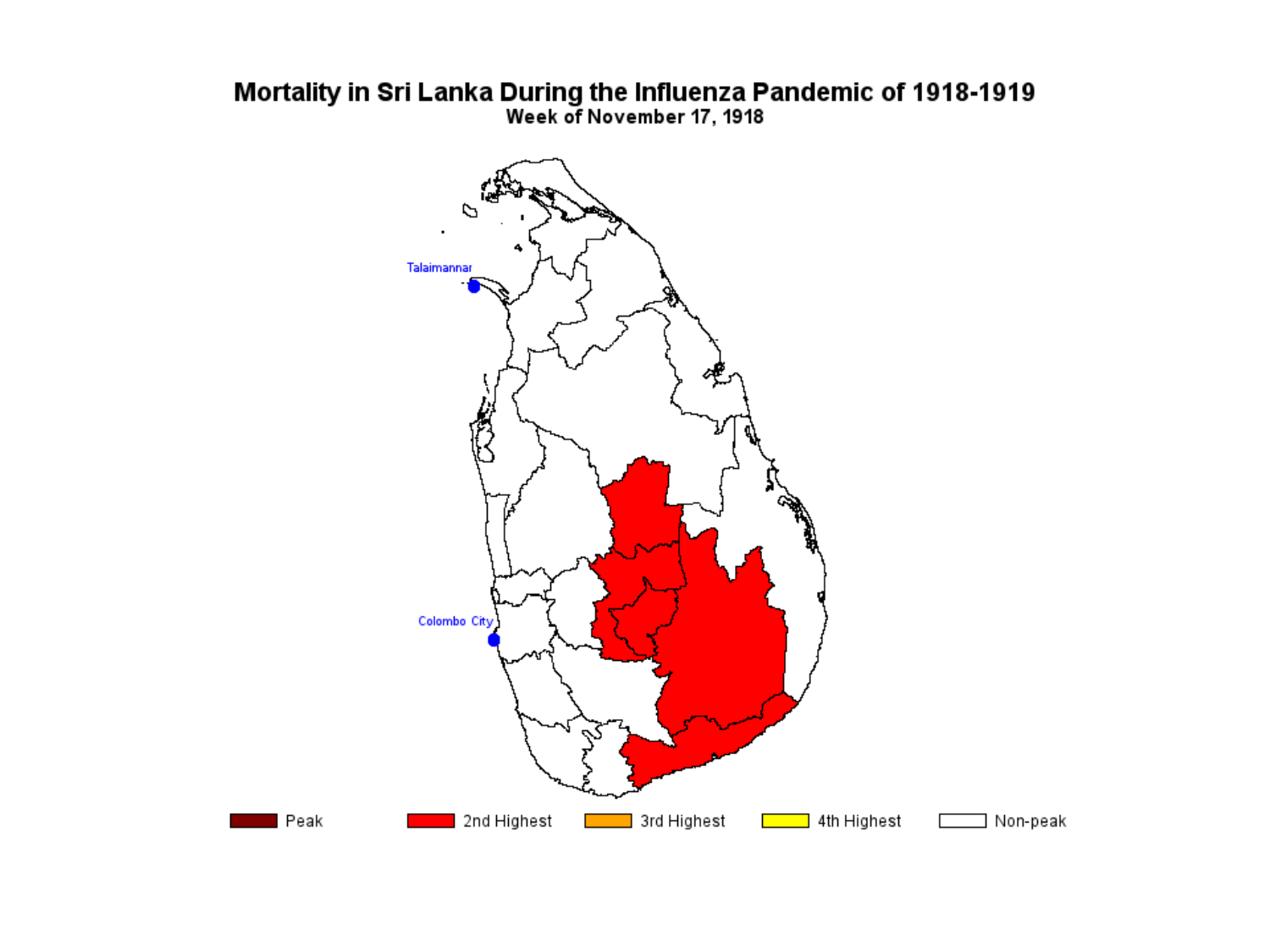

## Slide 13
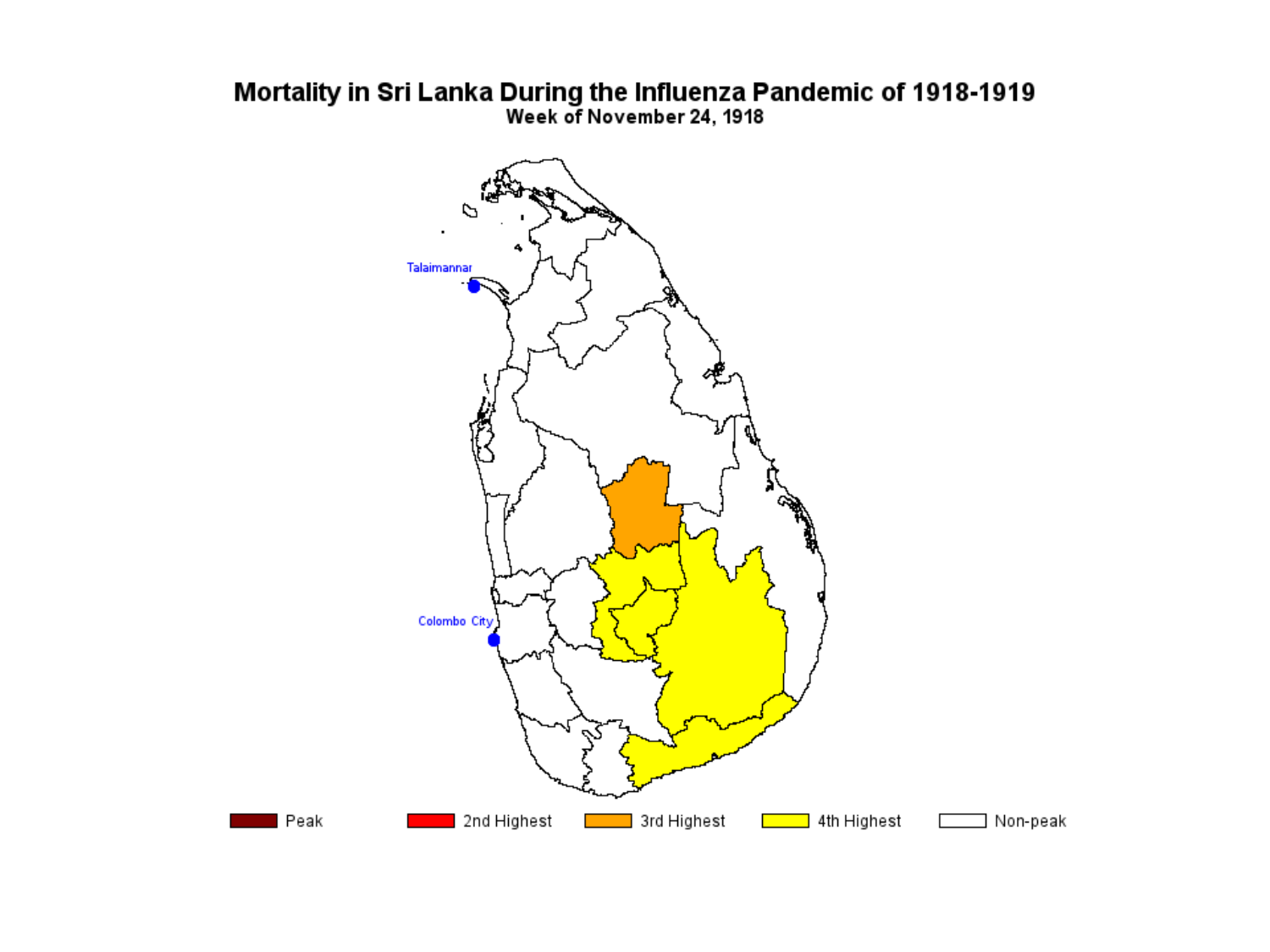

## Slide 14
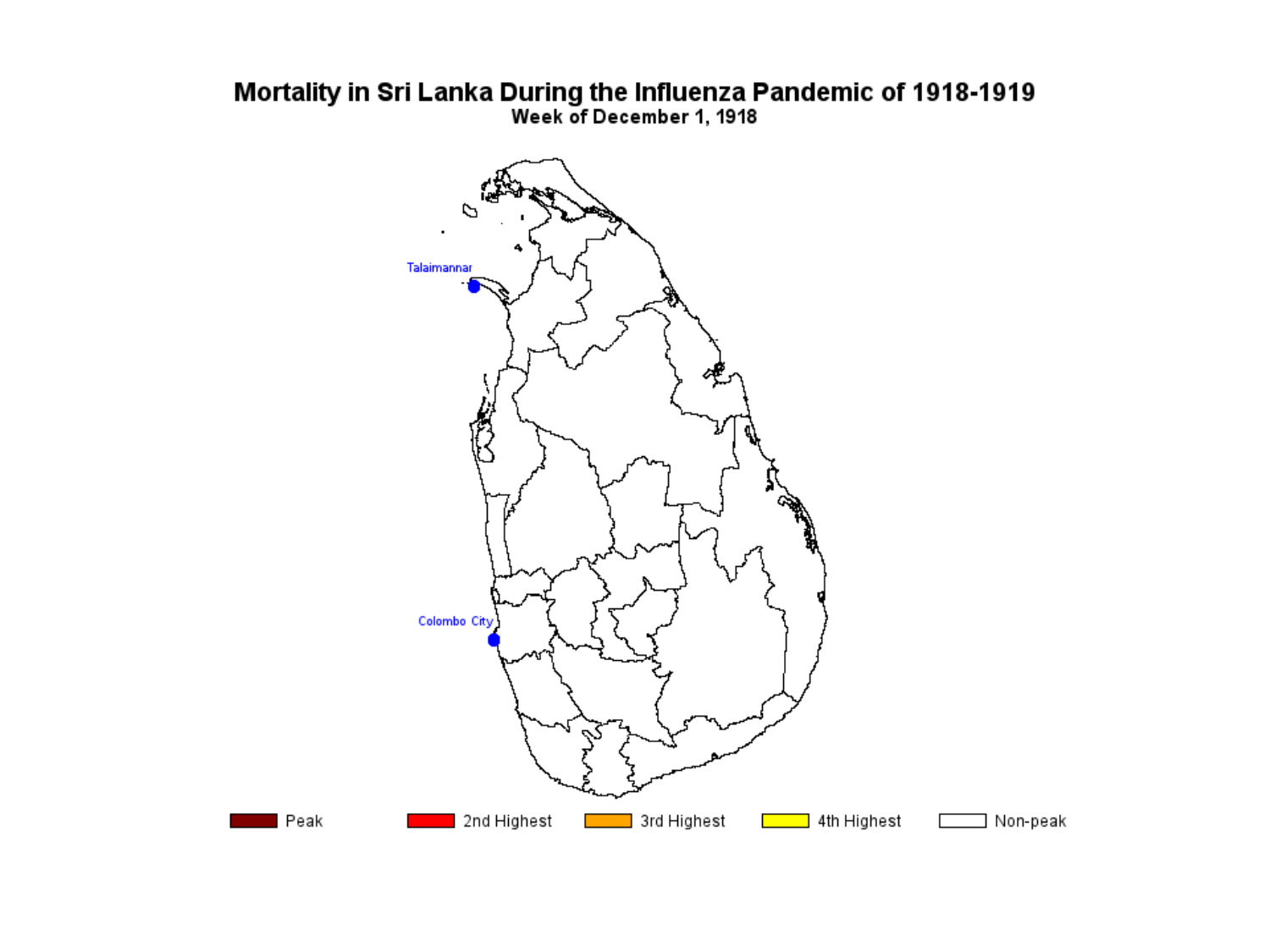

## Slide 15
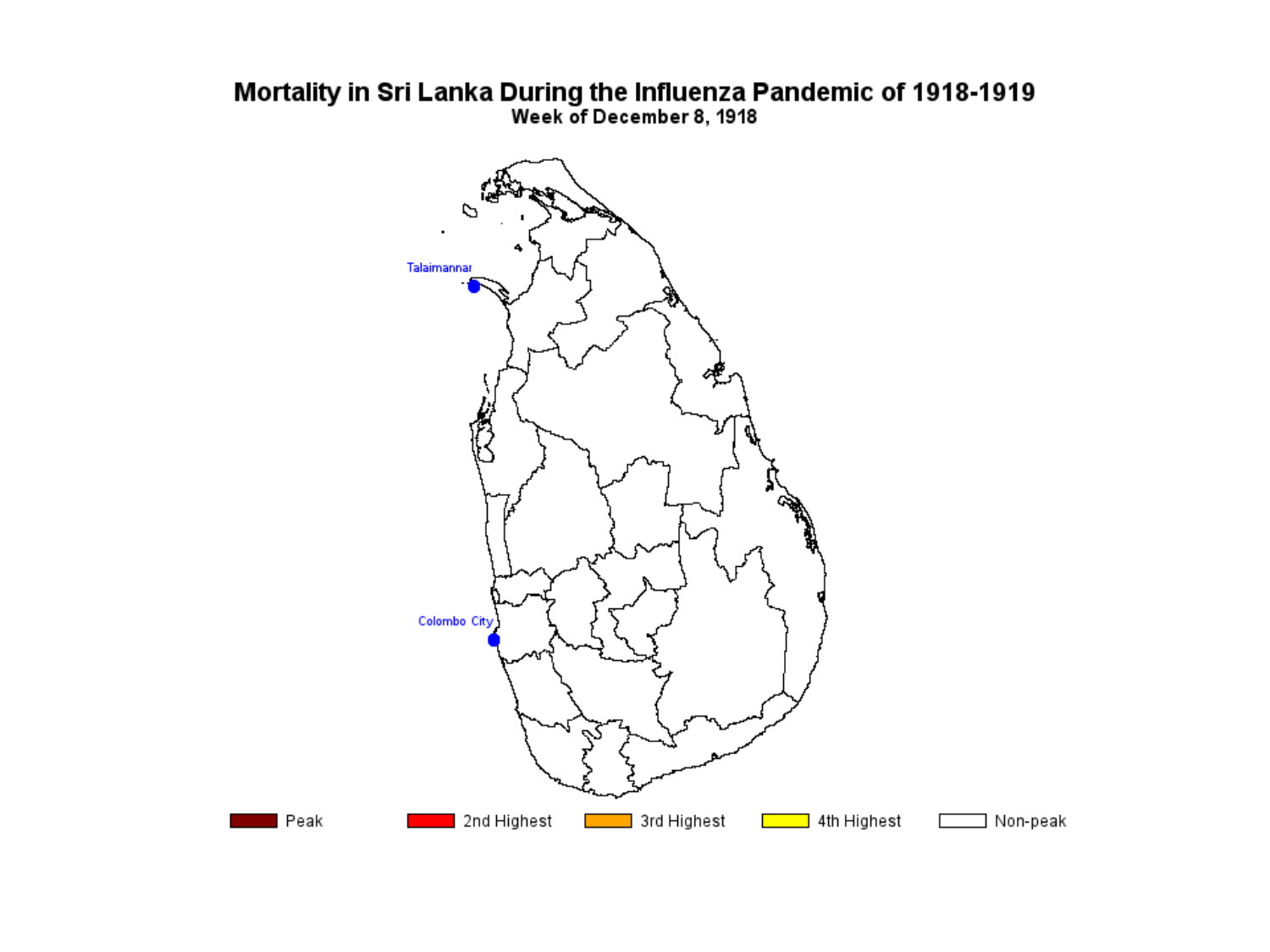

## Slide 16
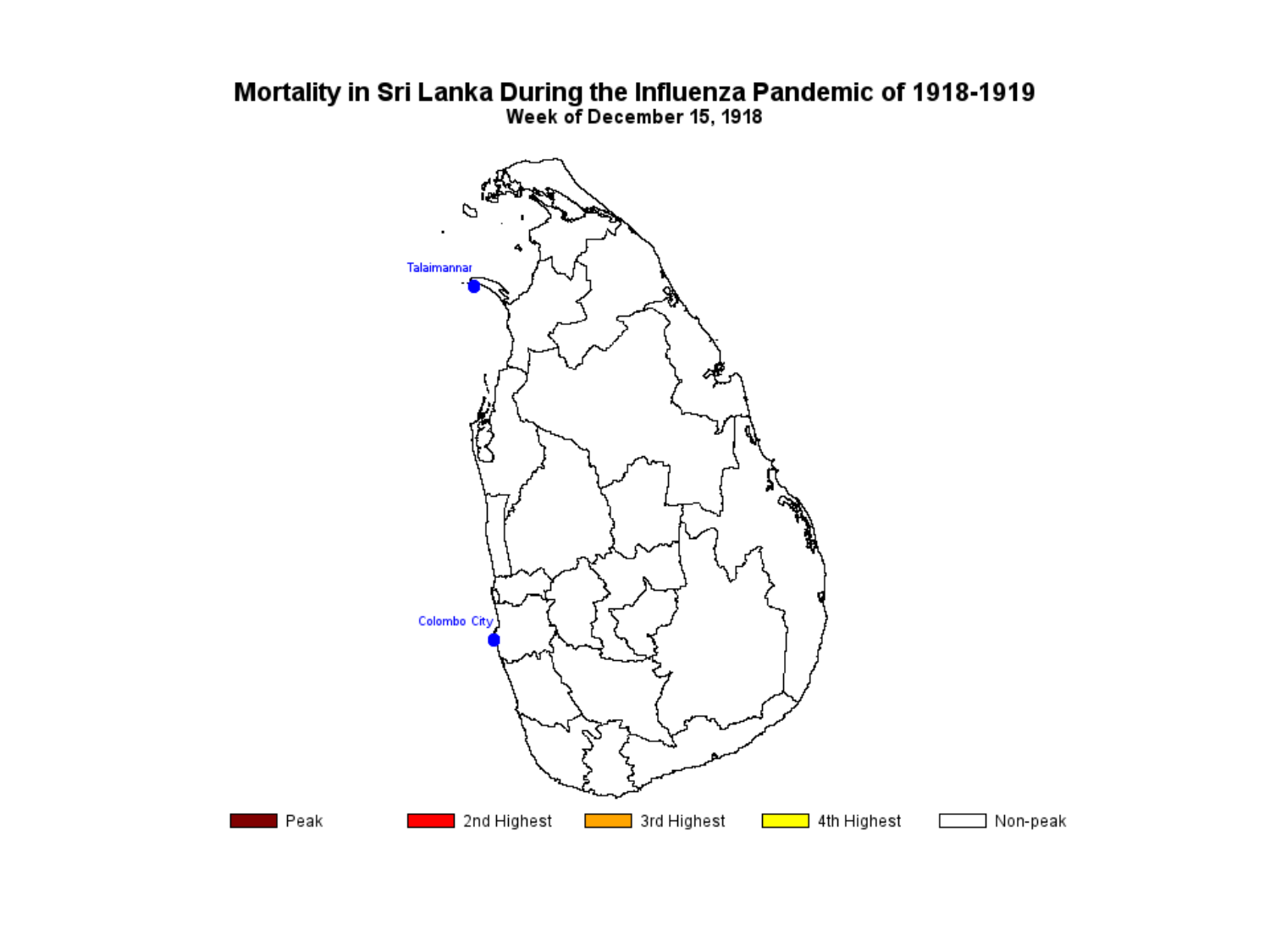

## Slide 17
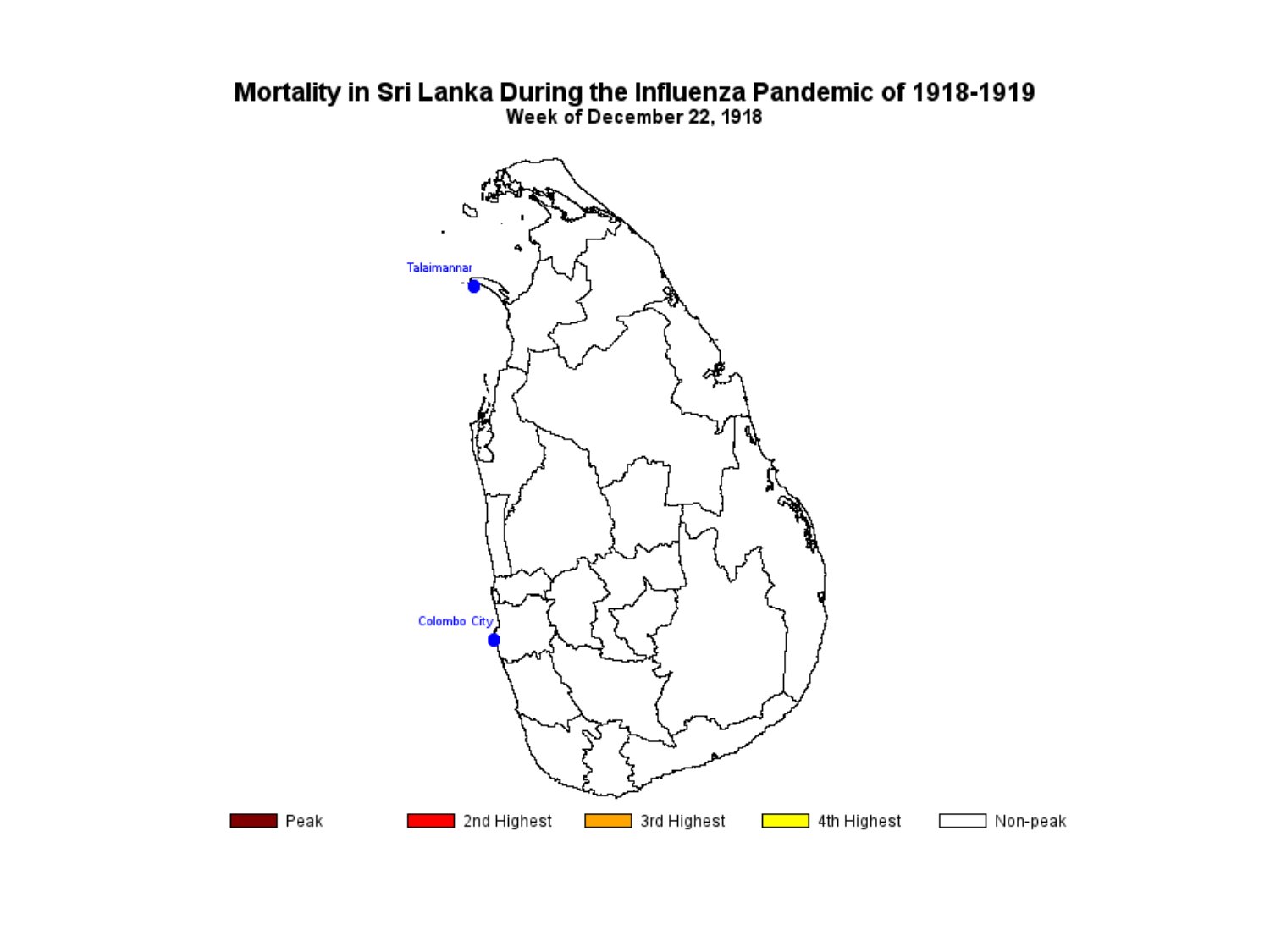

## Slide 18
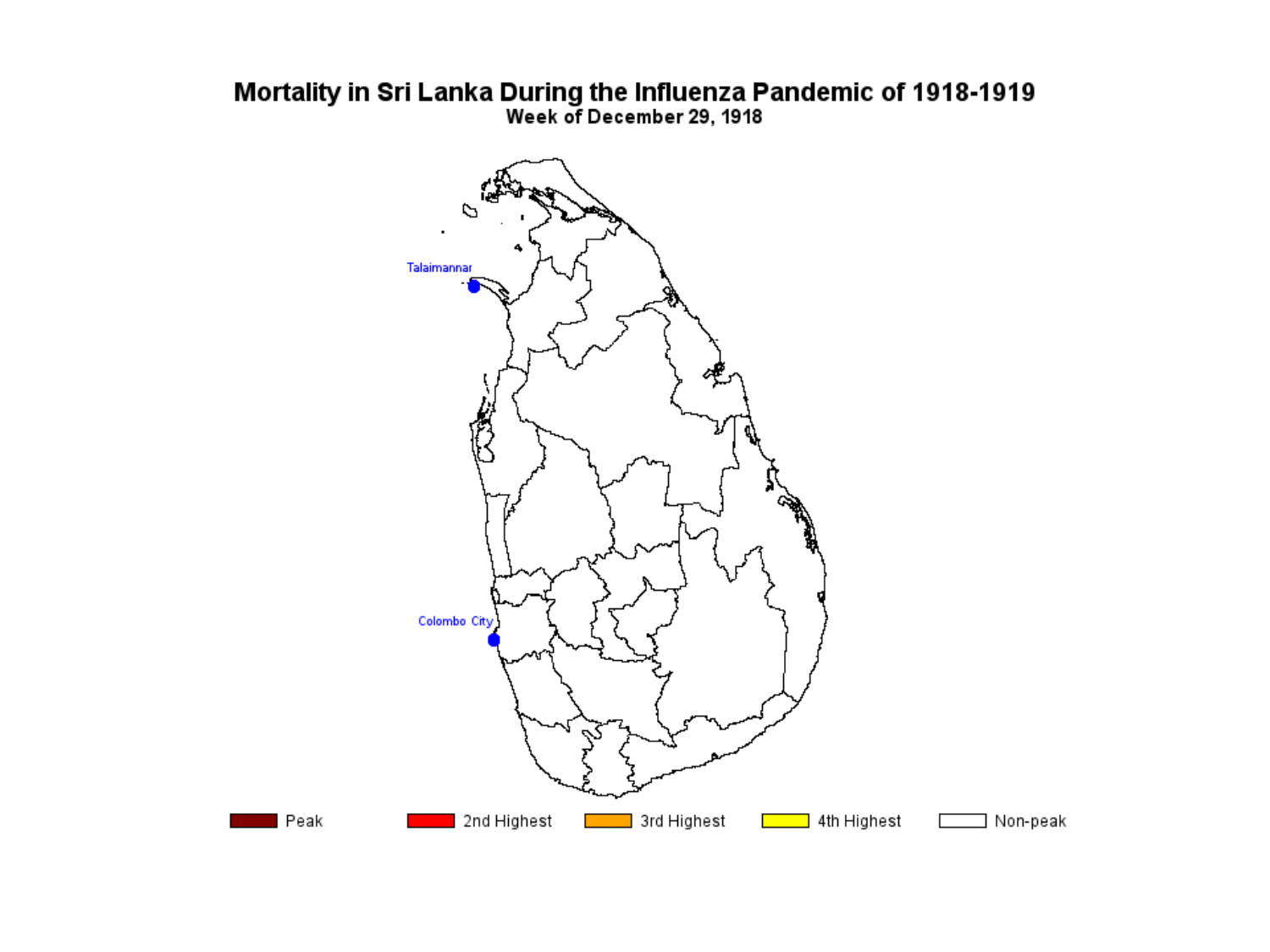

## Slide 19
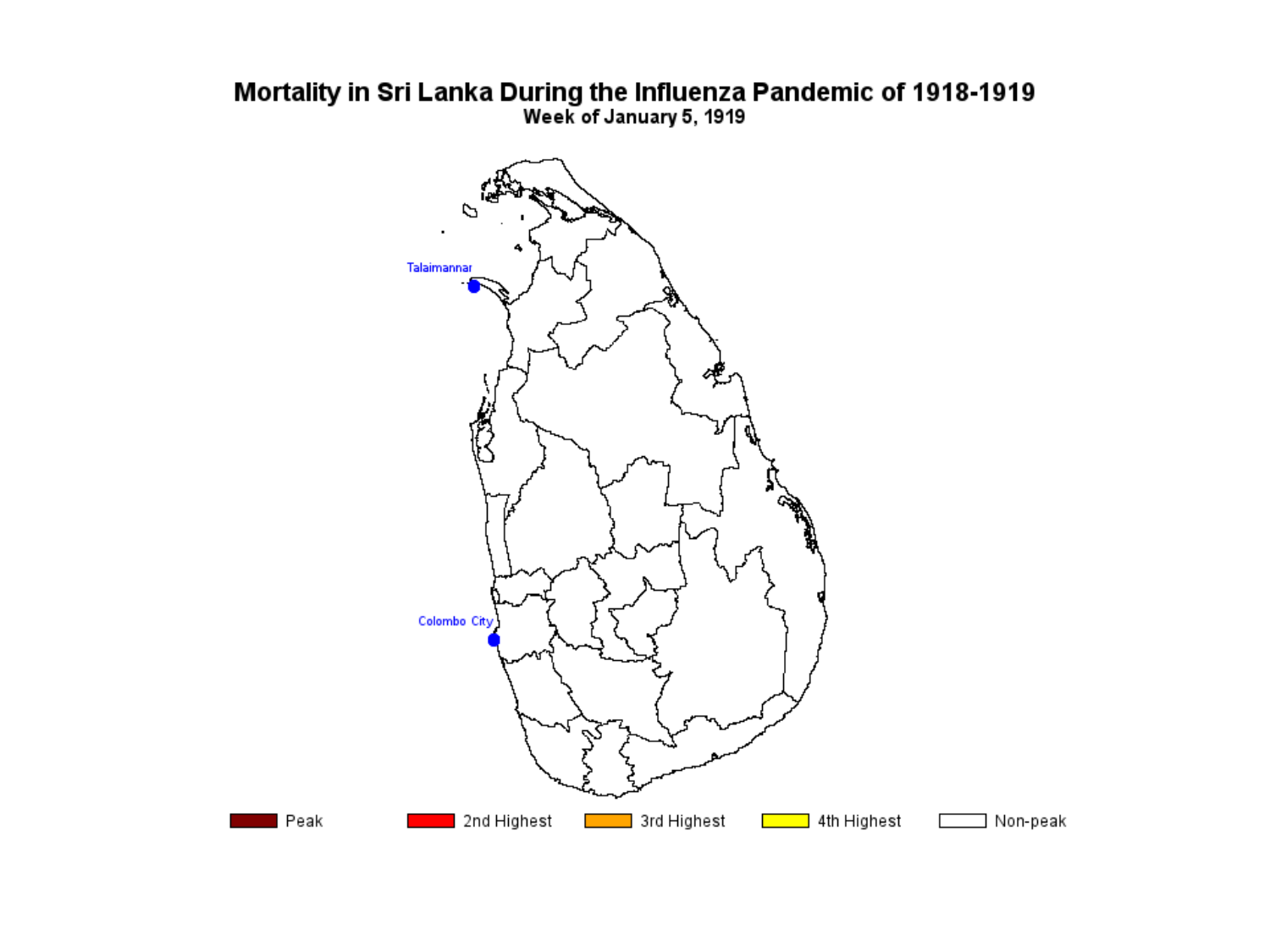

## Slide 20
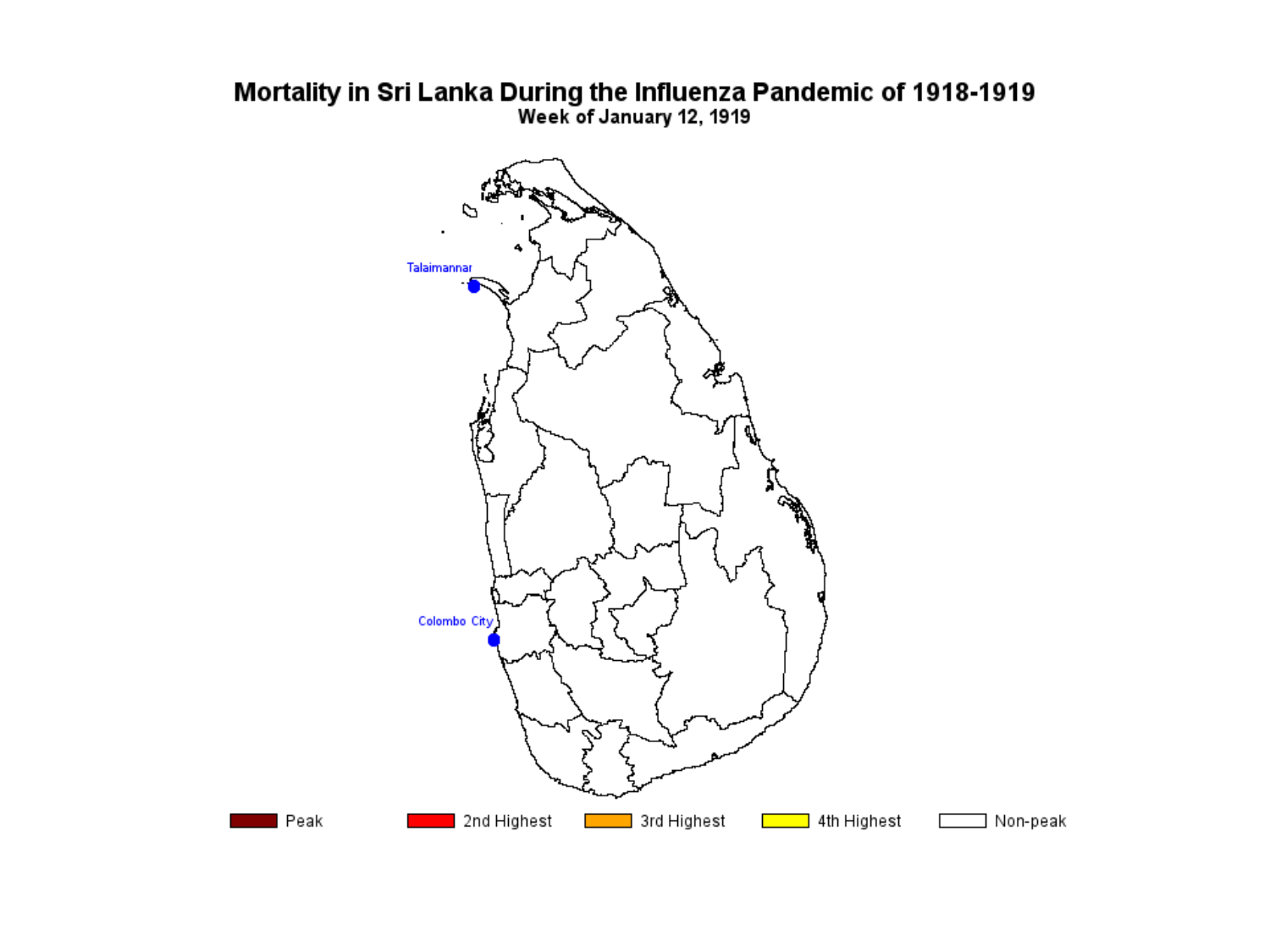

## Slide 21
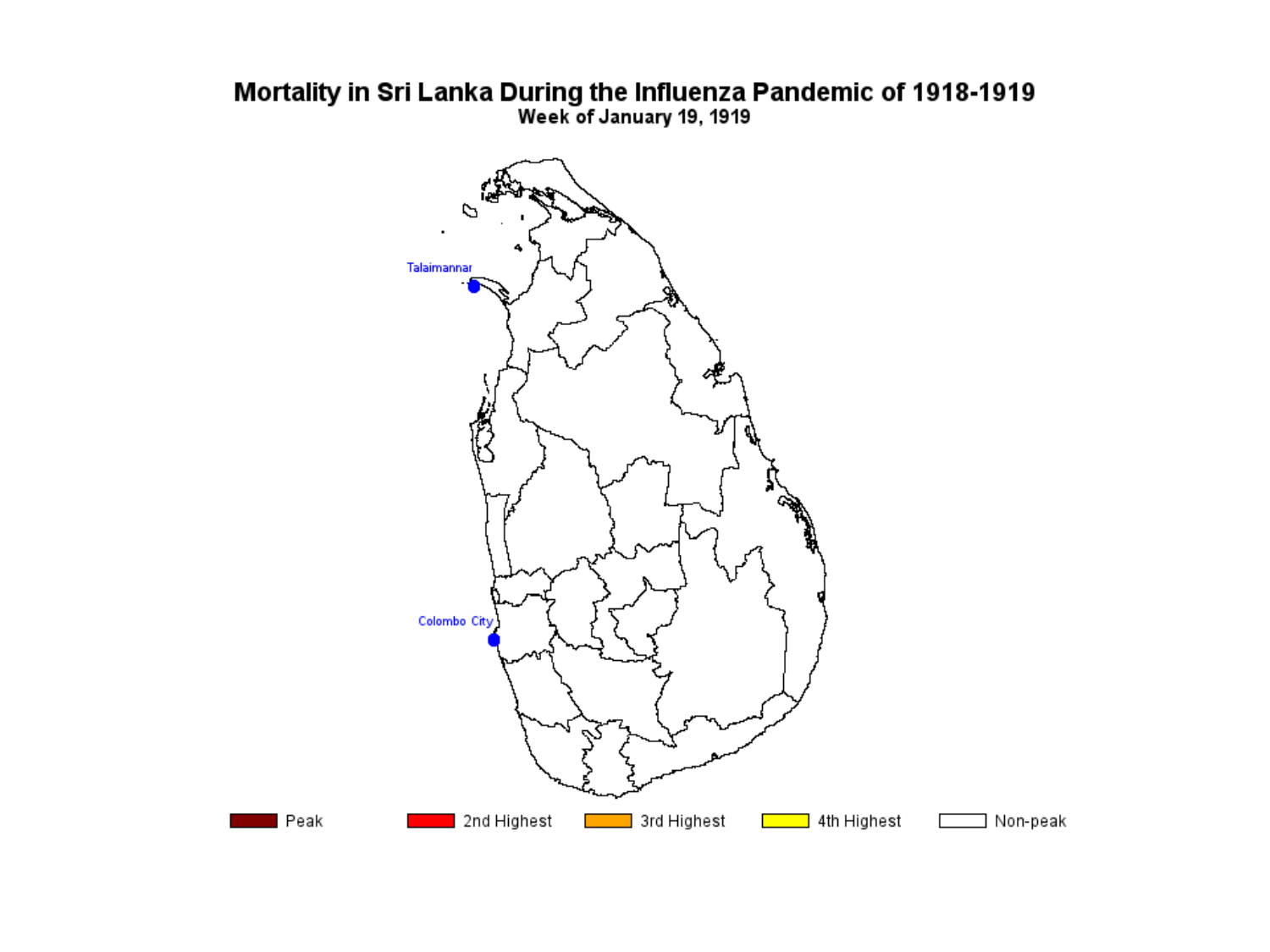

## Slide 22
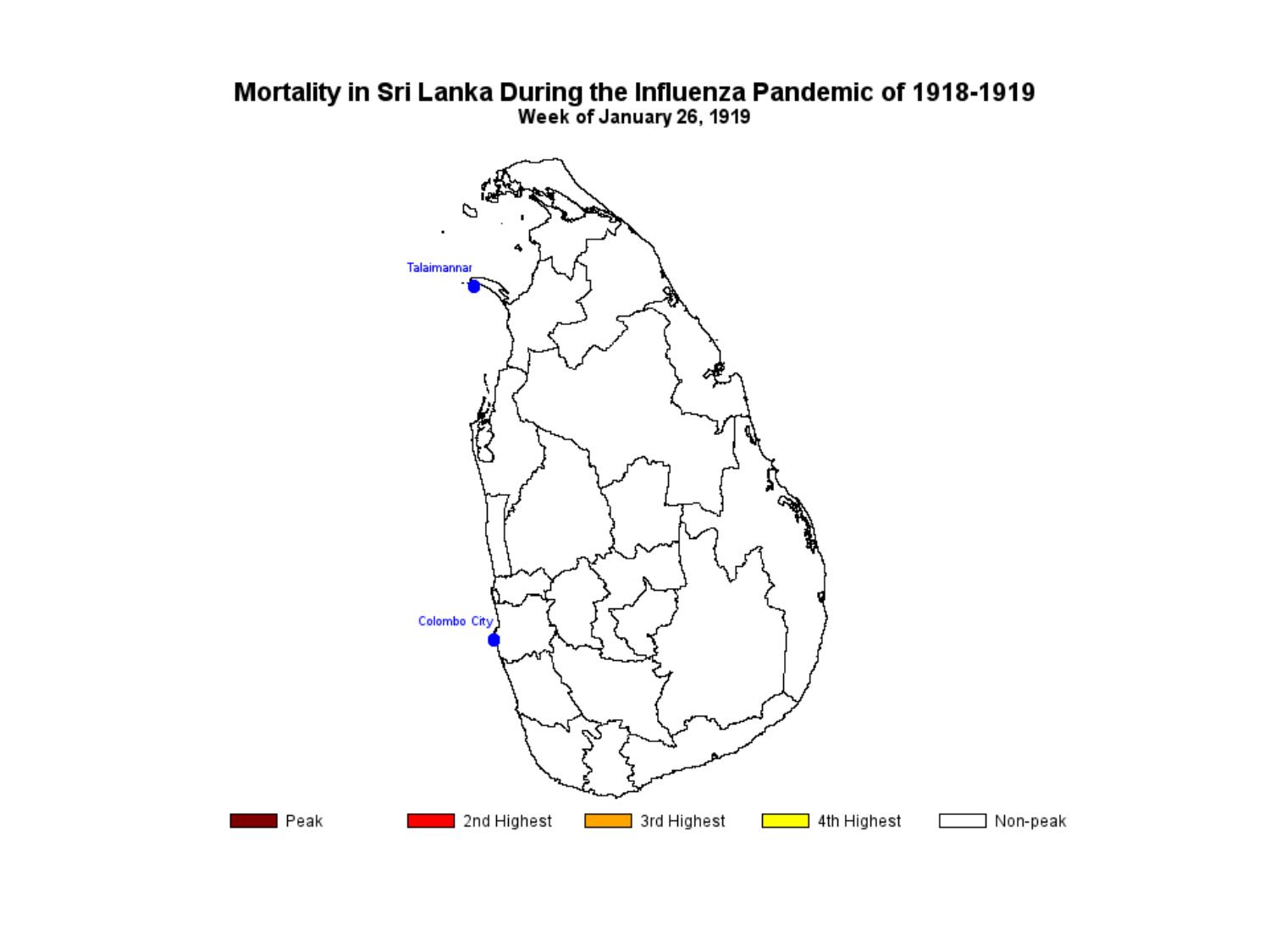

## Slide 23
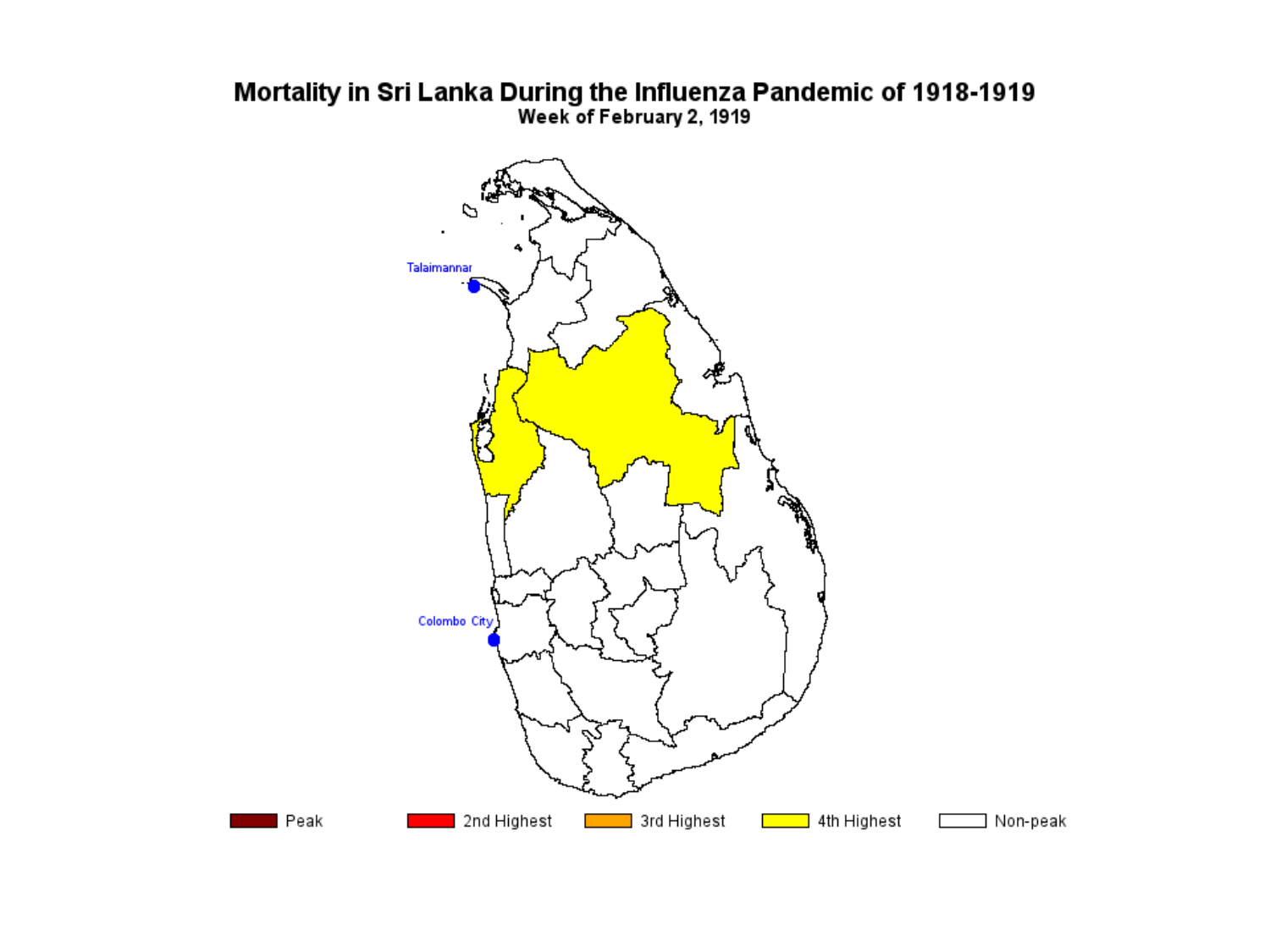

## Slide 24
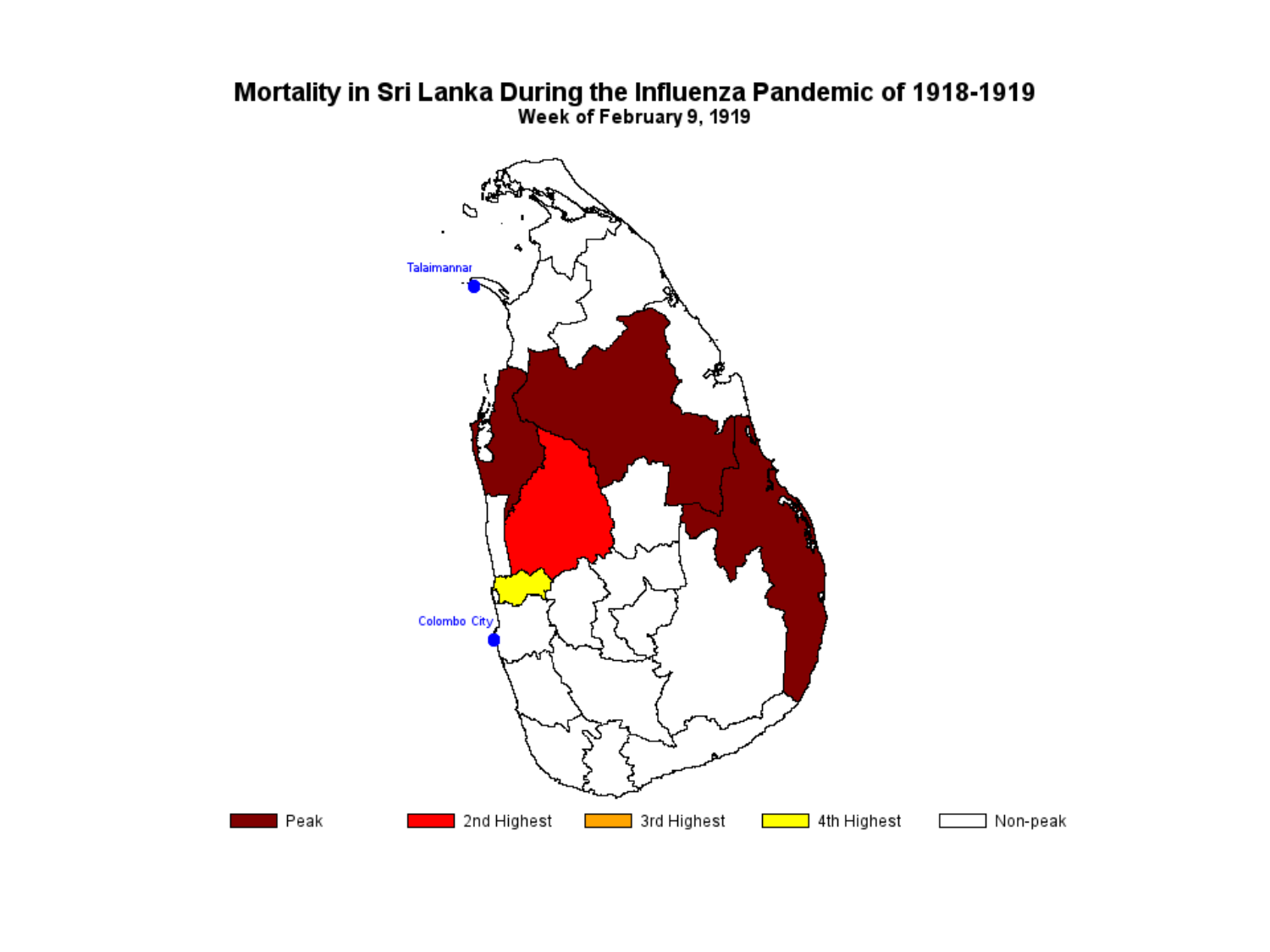

## Slide 25
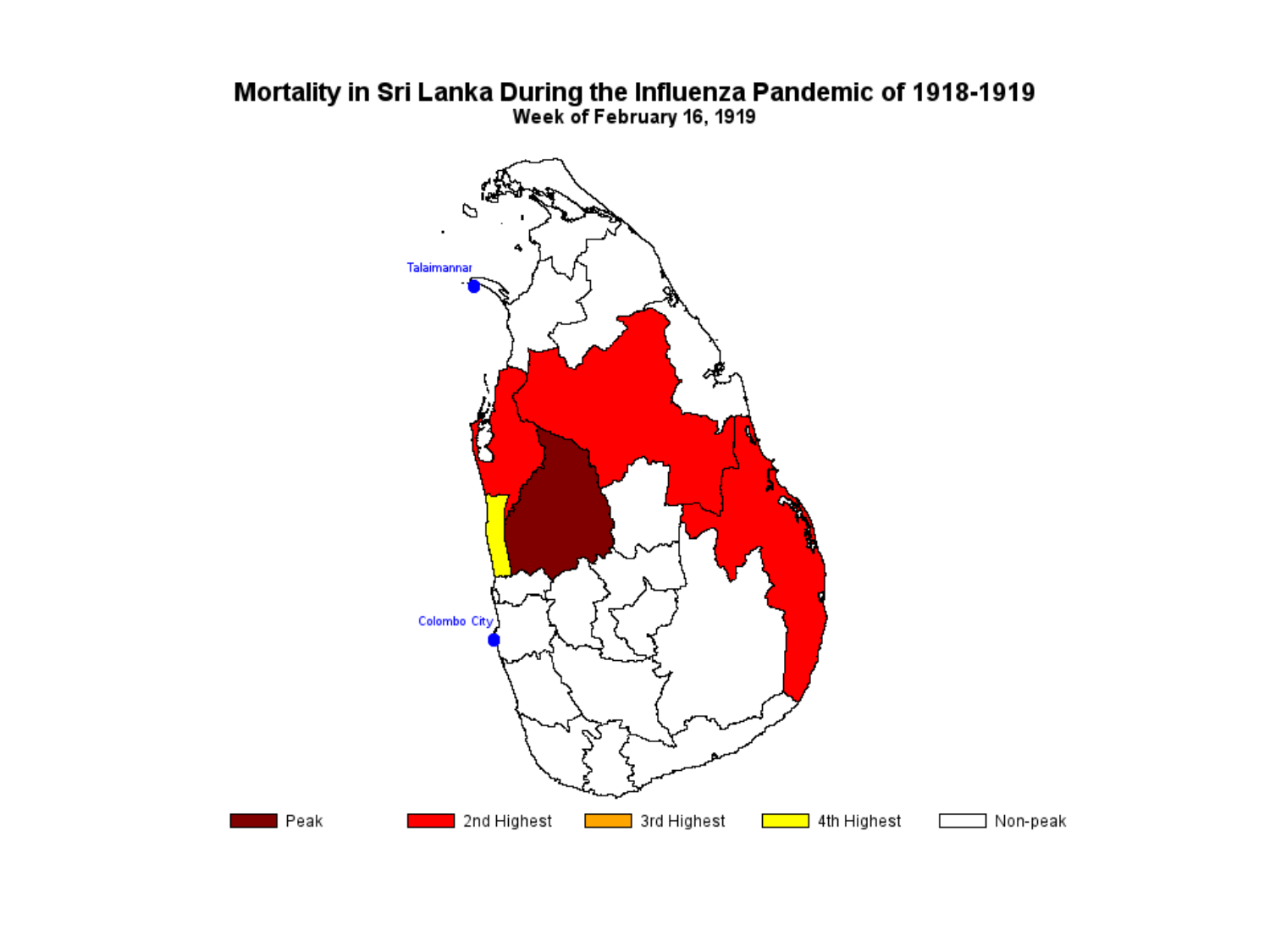

## Slide 26
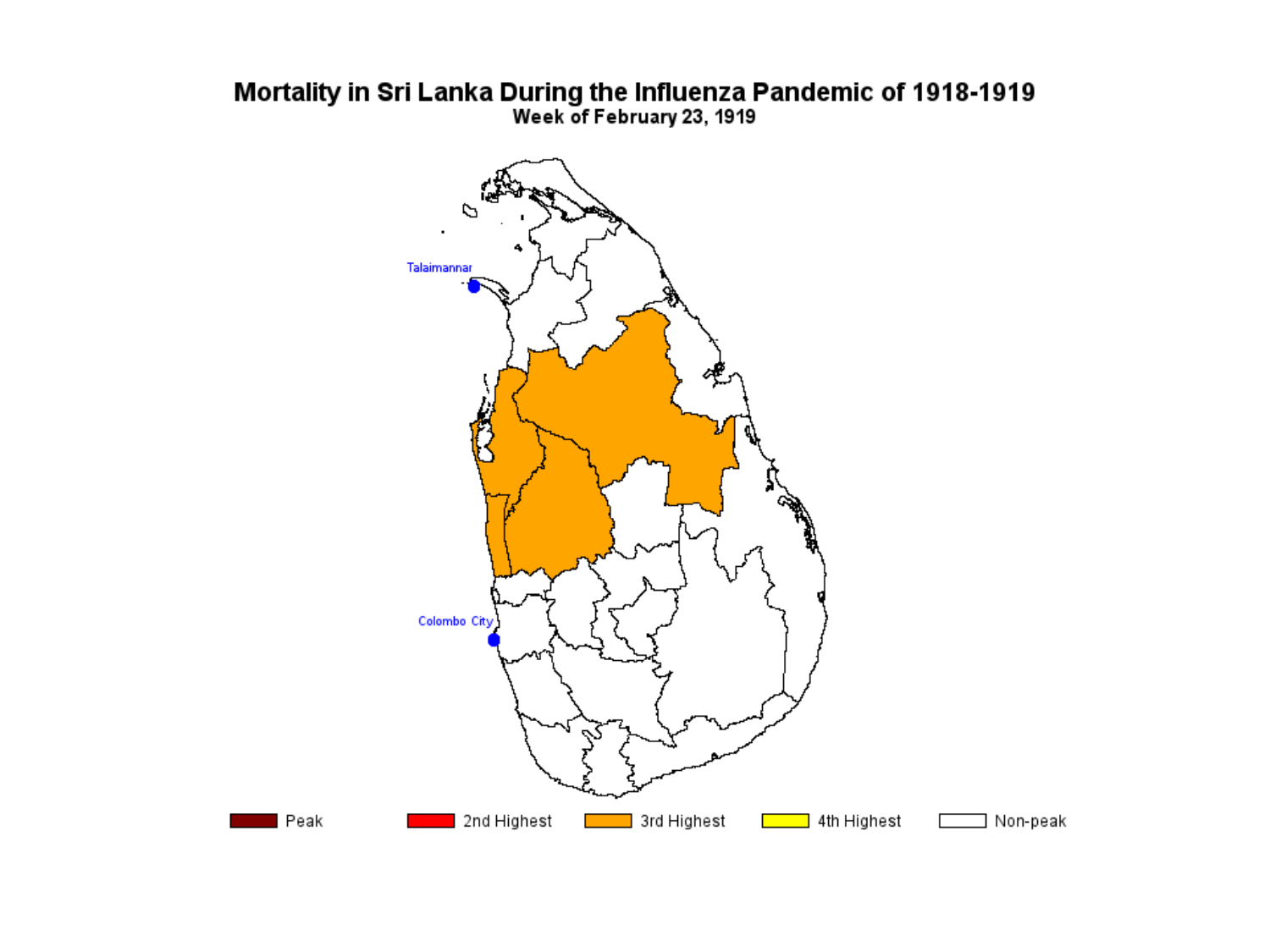

## Slide 27
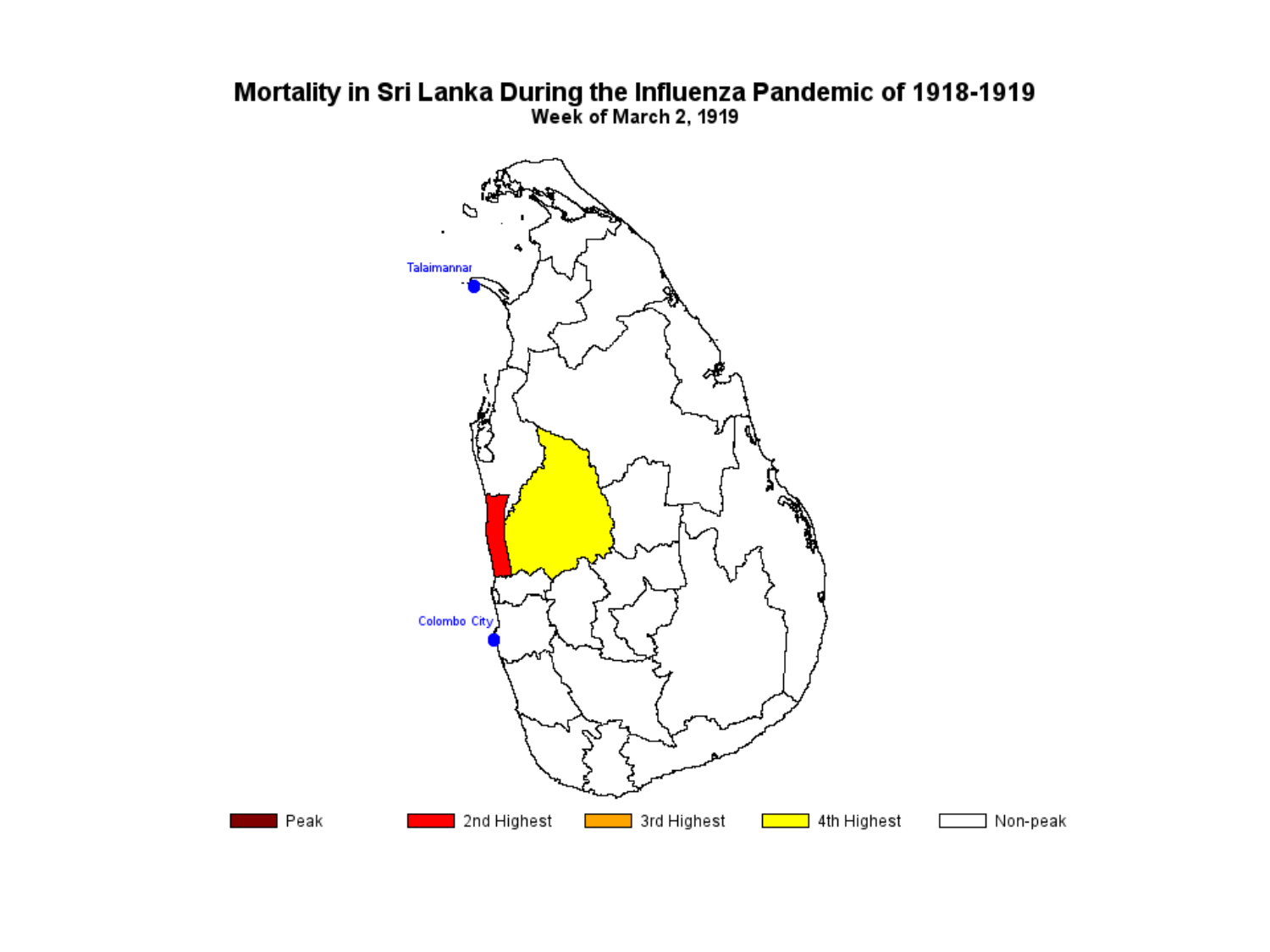

## Slide 28
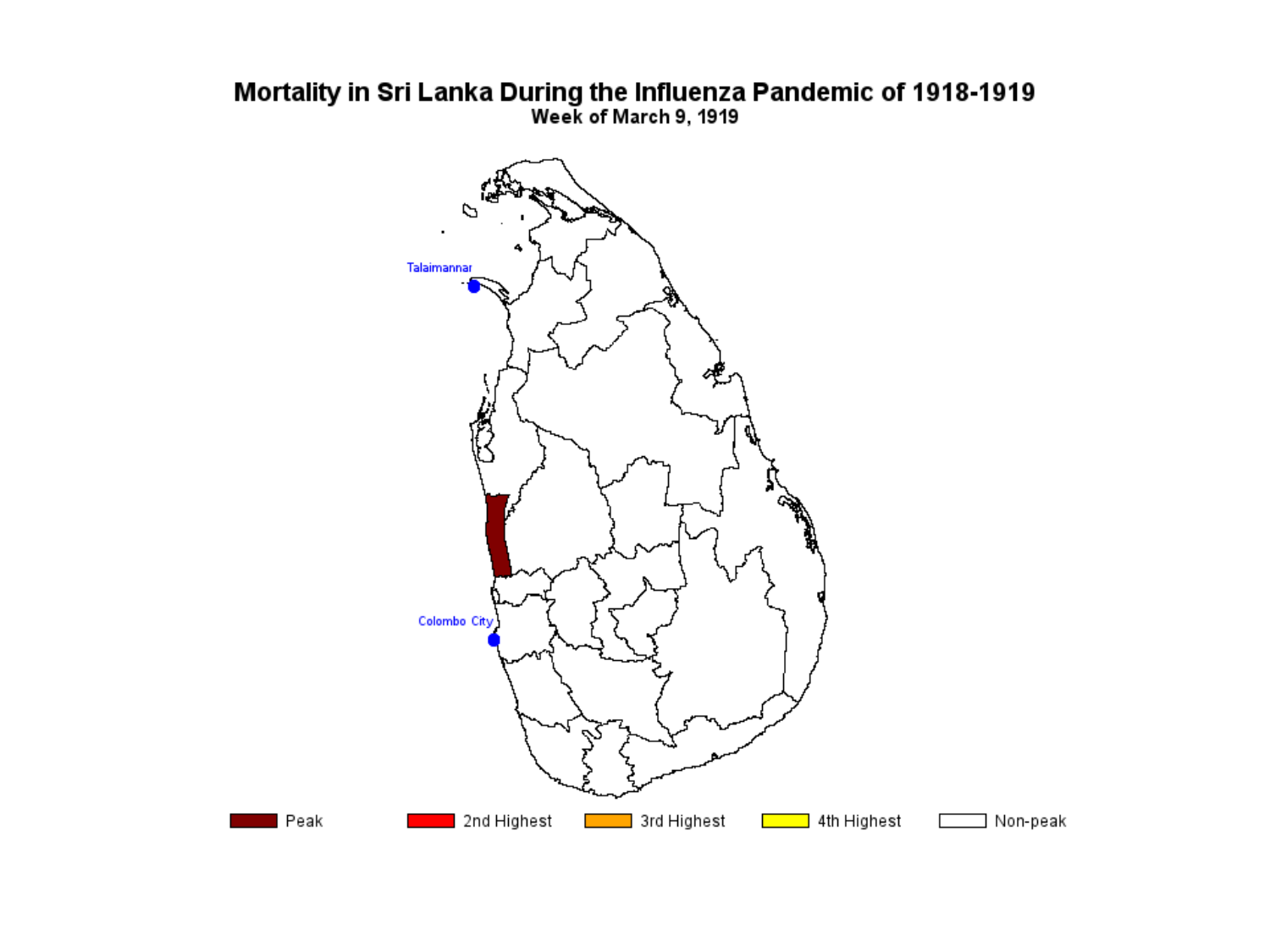

## Slide 29
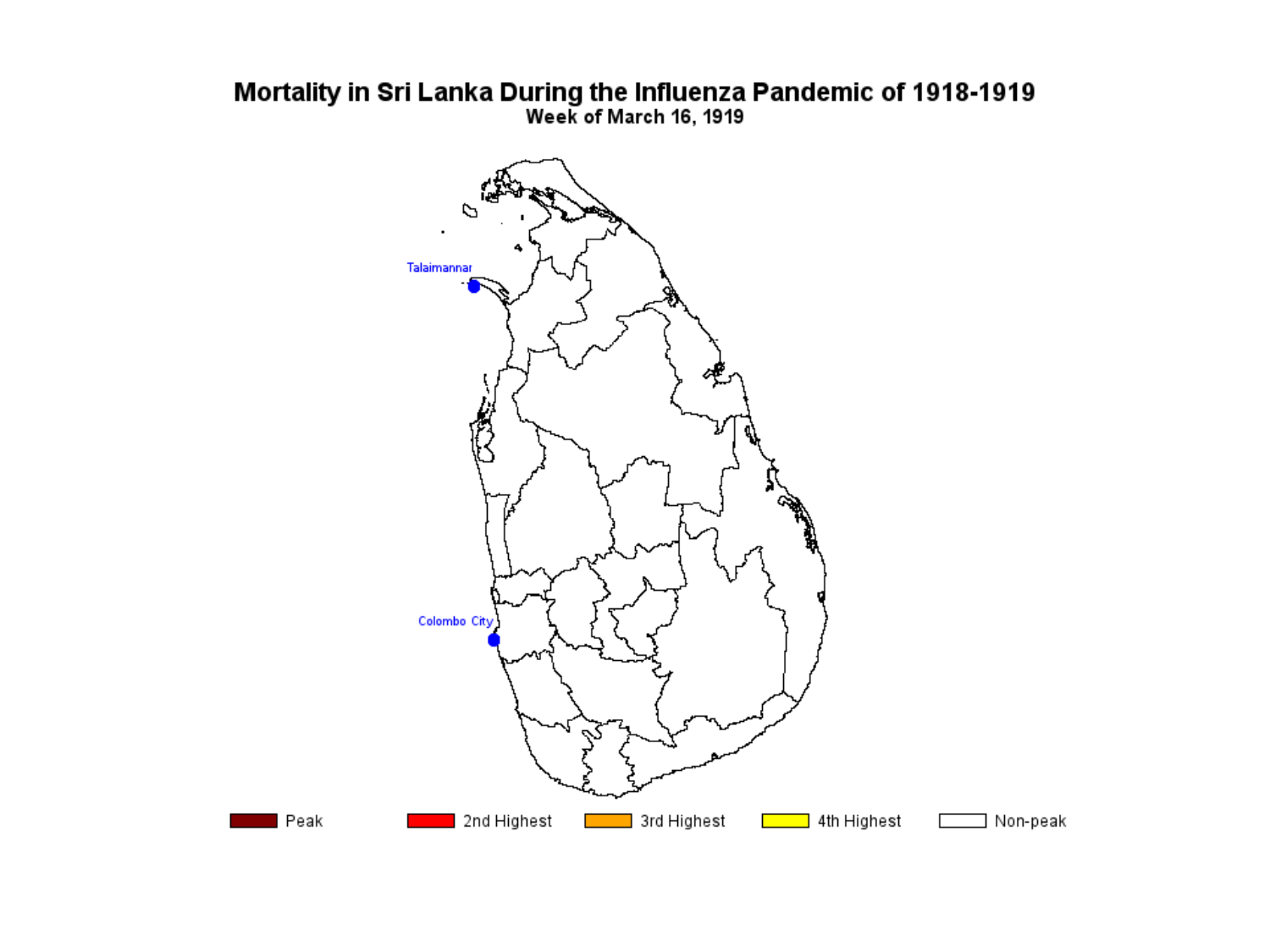

## Slide 30
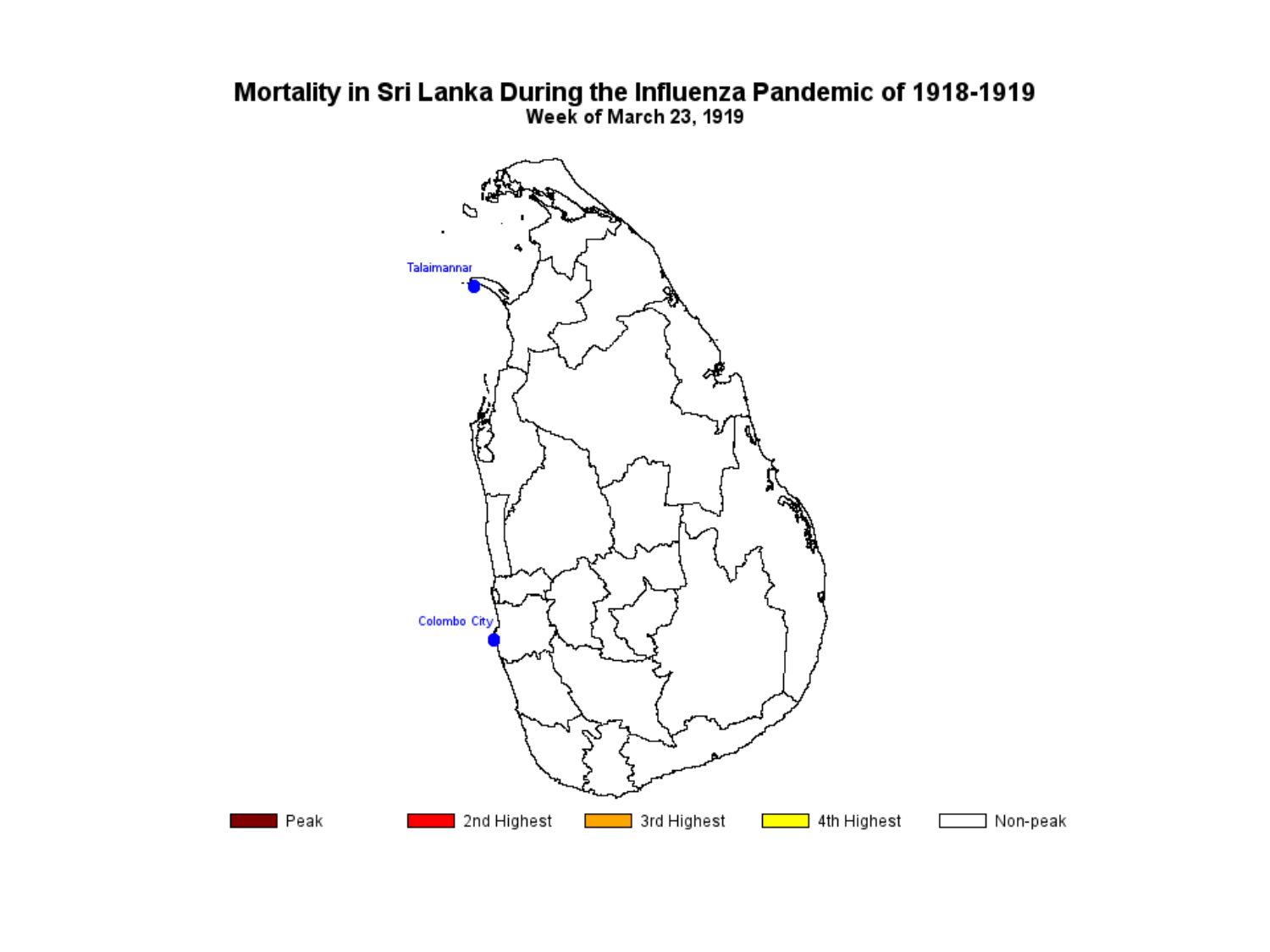

Supplement: Supplementary file 1 — Appendix S1. Weekly progression of influenza across Sri Lanka during the 1918-1919 pandemic. [file irv0008-0267-SD1.ppsx]
